# Examining performance and likelihood ratios for two likelihood ratio systems using the PROVEDIt dataset

**DOI:** 10.1371/journal.pone.0256714

**Published:** 2021-09-17

**Authors:** Sarah Riman, Hari Iyer, Peter M. Vallone

**Affiliations:** 1 Applied Genetics Group, National Institute of Standards and Technology, Gaithersburg, Maryland, United States of America; 2 Statistical Design, Analysis, Modeling Group, National Institute of Standards and Technology, Gaithersburg, Maryland, United States of America; National University of Sciences and Technology (NUST), PAKISTAN

## Abstract

A likelihood ratio (LR) system is defined as the entire pipeline of the measurement and interpretation processes where probabilistic genotyping software (PGS) is a piece of the whole LR system. To gain understanding on how two LR systems perform, a total of 154 two-person, 147 three-person, and 127 four-person mixture profiles of varying DNA quality, DNA quantity, and mixture ratios were obtained from the filtered (.CSV) files of the GlobalFiler 29 cycles 15s PROVEDIt dataset and deconvolved in two independently developed fully continuous programs, STRmix v2.6 and EuroForMix v2.1.0. Various parameters were set in each software and LR computations obtained from the two software were based on same/fixed EPG features, same pair of propositions, number of contributors, theta, and population allele frequencies. The ability of each LR system to discriminate between contributor (H1-true) and non-contributor (H2-true) scenarios was evaluated qualitatively and quantitatively. Differences in the numeric LR values and their corresponding verbal classifications between the two LR systems were compared. The magnitude of the differences in the assigned LRs and the potential explanations for the observed differences greater than or equal to 3 on the log_10_ scale were described. Cases of LR < 1 for H1-true tests and LR > 1 for H2-true tests were also discussed. Our intent is to demonstrate the value of using a publicly available ground truth known mixture dataset to assess discrimination performance of any LR system and show the steps used to understand similarities and differences between different LR systems. We share our observations with the forensic community and describe how examining more than one PGS with similar discrimination power can be beneficial, help analysts compare interpretation especially with low-template profiles or minor contributor cases, and be a potential additional diagnostic check even if software in use does contain certain diagnostic statistics as part of the output.

## 1. Introduction

Fully continuous probabilistic genotyping software (PGS) uses computer algorithms and complex calculations to apply biological, statistical, and mathematical models to resolve genotypes of contributors or assign evidential weight for the DNA typing results [1–4]. These models, unlike binary and semi-continuous models, use quantitative information contained within a profile (e.g. allelic designations, peak heights, molecular weights/fragment length), take into account stochastic effects, model peak height variability, and allow interpretation of low-level and complex DNA mixtures, therein reducing the need to infer using subjective reasoning [3, 5–10].

Numerous commercial [11–16] and open-source [17–21] software and freeware [22, 23] packages implementing fully continuous models have been developed. Differences exist among the programs in the way they model the distribution of allelic peak heights, stutter artifacts, mixture ratios, degradation, and stochastic events [8, 9, 24–28], though all use the same underlying genetic, physical, and chemical principles.

Most PGS require the assignment of two propositions, the prosecution proposition (H1) and defense proposition (H2) that include the specification of the number of contributors. Other parameters specific for each PGS are also required to deliver a key output, a Bayes factor, commonly referred to as the likelihood ratio (LR) [29, 30]. LR is the strength of the evidence in favor of H1 relative to H2. It is expressed as the ratio of two probabilities:

$$LR=\frac{Pr(E|H1,I)}{Pr(E|H2,I)}$$

where *E* is the findings or evidence and *I* is the relevant background information. The numerator is the probability of the findings given that H1 and background information are true and the denominator is the probability of the findings given that H2 and background information are true [31, 32].

So far there is no consensus within the forensic DNA community on implementing a standardized fully continuous PGS [33, 34]. As a result, depending on the software being used, the interpretation of the same DNA profile could yield different numeric LR values and, if used, different verbal characterizations [34, 35]. Even if the same PGS is used, the overall LR system could be different and hence will lead to different LRs [36–38]. Few studies explored the question of the degree of variability in LR values across various fully continuous PGS [39–42]. These studies were based on limited number of samples, did not quantify the differences in LRs, and concluded that the models yielded similar LRs despite the differences among the PG modeling assumptions. Only in [34], the authors demonstrated the impact of inter-model variability on numerical values and verbal expression of the LRs of H1 true cases when four variant models of the same continuous software, CEESIt, were compared.

To further understand the amount of variability expected when mixtures are interpreted using different systems, we here performed large-scale comparison and assessed the LR values produced by two reputable and well-cited fully continuous PG models. For this illustration, we chose STRmix v2.6 (a commercial software that uses the Bayesian approach) and EuroForMix v2.1.0 (an open-source software that uses the maximum likelihood estimation (MLE) method) [11, 17]. A large dataset of ground truth known 2-person, 3-person, and 4-person mixture profiles was selected from the publicly available PROVEDIt database [43, 44]. We first investigated the discriminating power of the two LR systems using Receiver Operating Characteristic (ROC) plots to ensure that we are not comparing two PG models with substantially different discriminating performance. We then quantified the differences in the log_10_(LRs) assessed by the two systems in H1 true cases as well as in H2 true cases and evaluated the possible reasons behind these discrepancies. Various decisions made as to the choice of thresholds and software parameter settings are outlined in detail in the methods section.

We believe that this is the first study that is large-scale, uses publicly available data, and evaluates the extent to which different models disagree (e.g. by a factor of 10, factor of 100, more than a factor of 1000). We outline the steps that may be used by other laboratories to assess the performance of different LR systems and analyze the resulting data. We also share the generated LR values in the interest of transparency and literature-to-literature comparisons by other researchers. Notably, the results are expected to vary if other parties conduct a similar analysis but use different software versions and protocols. Nevertheless, the process of comparison will essentially consist of the same steps outlined herein.

## 2. Methods

### 2.1. PROVEDIt dataset description

In this study, the Short Tandem Repeat (STR) profiles used to set the PGS parameters and calculate the LRs were selected from the PROVEDIt (Project Research Openness for Validation with Empirical Data) dataset that was amplified with GlobalFiler (GF) kit (29 cycles) and analyzed on 3500 Genetic Analyzer with an injection time of 15 seconds (s) [43, 44]. Both raw (.hid) and filtered (.CSV) files were used in the analysis. The filtered files present in the PROVEDIt database consist of the exported genotype tables containing allele designation, base pair (bp) size, and peak heights information for each sample profile analyzed in GeneMapper ID-X at an analytical threshold (AT) of one Relative Fluorescent Unit (RFU). Also, these filtered files did not contain artefacts such as pull-up, minus A, and– 2 bp in the SE33 locus as they were removed according to a defined criteria set by Alfonse et al. [43].

A total of 154 two-person (2P), 147 three-person (3P), and 127 four-person (4P) mixture profiles were obtained from the filtered (.CSV) files and used for LR calculations. The 2P, 3P, and 4P testing sets were prepared using DNA from 22 individuals for whom reference profiles were also available. The profiles used had varying: (1) minor contributor template amounts, (2) total input template amounts, (3) contributor ratios, and (4) DNA quality. A detailed description of the 2P, 3P, and 4P profiles that were used in the study is shown in S4 Table.

The mixture input files were analyzed using the per dye specific ATs discussed in Section 2.3 (shown in Table 1) and converted along with person of interest (POI) files into a format specific to each software [45, 46]. Non-numeric values, Off-Ladder “OL” peaks, were eliminated from all the analysis [46].

**Table 1:** Summary of STRmix v2.6 and EFM v2.1.0 interpretation parameters and reported LR values.

Software	Interpretation summary
STRmix v2.6 https://www.strmix.com/	• Per dye ATs were set in STRmix kit settings (Blue = 35; Green = 65; Yellow = 45; Red = 50; Purple = 60)
	• Drop-in frequency = 0.0015 and drop-in cap = 180 RFU
	• MCMC settings: 8 chains of 100,000 burn-in accepts, 50,000 post burn-in accepts per chain
	• N-1, N-2 and N+1 stutter peaks modeled
	• 333 single source profiles used for Model Maker
	• Allelic variance (α, β) 5.653, 2.961; back stutter variance (α, β) 1.501, 27.227; forward stutter variance (α, β) 1.501, 31.710; double back stutter (α, β) 1.771, 21.655; LSAE variance 0.031
	• Sub-source LR values labeled as sub-source LRs in STRmix report were considered
EuroForMix v2.1.0 http://www.euroformix.com	• Overall lowest AT value was set (35 RFU)
	• Drop-in probability = 0.0015 and Drop-in hyper-parameter (λ) = 0.032
	• N-1 stutter peaks modeled
	• Degradation and stutter models jointly selected
	• Sub-source LR values labeled as MLE based LRs in EFM reports were considered
Both software	• Same/fixed mixture EPG features
	• Input mixture profiles were analyzed using the per dye ATs (Blue = 35; Green = 65; Yellow = 45; Red = 50; Purple = 60)
	• Same defined pair of propositions
	• Same combination of comparisons (mixture vs POI) per each analysis
	• True NOC
	• NIST 1036-Caucasian allele frequencies [57]
	• *F*_ST_ *(*θ*)* = 0.01 [28, 58]

### 2.2. The LR system

**Fig 1:**
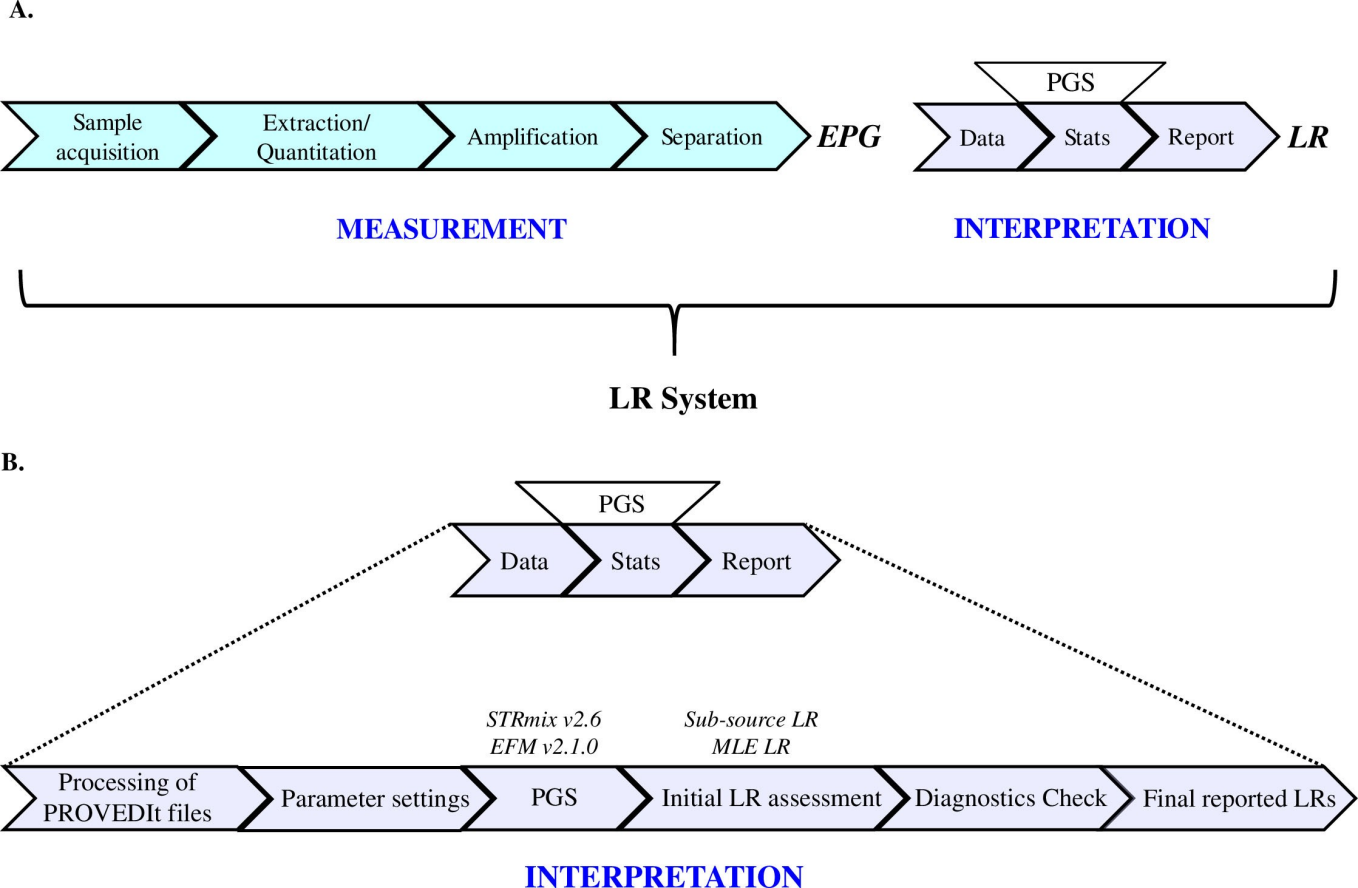
Schematic overview of the Likelihood Ratio (LR) system, adapted from [47, 48].

The conventional CE genotyping workflow used in forensic DNA laboratories is composed of several steps that can be grouped into two processes: measurement and interpretation (Fig 1A) [47]. The measurement process involves genomic DNA extraction, quantification, amplification using commercial multiplex STR kits (herein GF 29 cycles), and electrophoretic separation (herein 3500 at 15s injection time). The outcome of the measurement process is an electropherogram (EPG) composed of the length variants, heights, and sizes of the allelic and non-allelic peaks. The interpretation process involves data analysis. The outcome of the interpretation process is a strength of evidence statement often reported in the form of a LR and typically requires PGS.

Our definition of the LR system is the entire pipeline starting from sample acquisition all the way to LR calculation. The PGS is a piece of the whole LR system. Therefore, performance assessment of the LR system is not only an assessment of the software but an assessment of the entire process.

Herein the measurement process was established by Alfonse et al. [43] as mentioned previously in Section 2.1 and therefore was fixed for both LR systems. Thus, the performance assessment in this study encompasses the interpretation process as shown in Fig 1B that includes:

- our decision of using and processing the filtered PROVEDIt files
- parameter values determined according to the chosen software (discussed below in detail)
- the choice of PGS (herein STRmix v2.6 and EFM v2.1.0)
- the initial assessment of the LR values
- the check of diagnostics (review of per locus LR, deconvolution, genotypic weights, Gelman-Rubin statistics, log likelihood, and model selection)
- the reporting of the LRs

### 2.3. Analytical Thresholds (ATs)

To determine the AT, 41 pristine single source DNA profiles with varying amounts of DNA template 0–0.5 ng were obtained from the filtered version (.CSV) of PROVEDIt files [44]. The list of the 41 samples selected for AT determination are detailed in S1 Table. Allelic, stutter, and other artifactual peaks were discarded from these profiles. The mean (μ) and standard deviation (σ) of the remaining peaks (noise observations) were estimated per dye-color channel. Then, AT was determined by substituting the values of μ and σ in the following equation: AT = μ + k* σ, where k was set to 10 [36, 49–52]. The AT values were rounded up to the nearest multiple of 5 (Table 1) [36]. All peaks in the input profiles explored in this study with peak heights below the determined dye-specific AT values (shown in Table 1) were filtered out before importing the data into STRmix and EFM.

Dye-specific ATs were set in STRmix as determined empirically and shown in Table 1. EFM v2.1.0 allows the user to set an overall single AT value [45]. The lowest RFU value (35 RFU) was used as the AT parameter in the EFM software.

### 2.4. Drop-in

Raw data (.hid files) of a set of 189 negative control profiles (listed in S2 Table) from PROVEDIt database were analyzed in GeneMapper ID-X v1.5 with a 35 RFU for all the dye channels. The selected profiles that were amplified with no DNA (0 ng) resulted in 7 drop-in events.

For STRmix, the drop-in frequency and drop-in cap parameters were determined and entered in the software. The frequency of the observed drop-in events was determined by using the instructions contained in the drop-in worksheet available on STRmix support website [53]. The highest drop-in peak observed in this study was 101 RFU. To set the drop-in cap at a value that is greater than the 101 RFU as recommended in [36, 53], we calculated the μ and σ of the peak heights of the observed drop-in events and substituted these values in the following equation: Drop-in cap = μ + k* σ, where k was set to ≈ 5. The drop-in cap was then rounded up to the nearest multiple of 5. The determined values of drop-in frequency and drop-in cap are shown in Table 1. Due to the few drop-in events (only 7) observed within our analysis a uniform distribution was selected in the software [46, 53].

EFM requires the setting of the drop-in parameters, the drop-in probability (C), and the hyper-parameter (λ). Here, C was determined using C = n/N*L, where C is the drop-in probability per marker, n is the number of drop-in events, N is the number of samples used to count the number of drop-ins, and L is the number of markers in each sample used to count number of drop-ins. The estimated λ was determined using λ = n/∑_i_(x_i_ -T), where T is the analytical threshold used for analyzing drop-in, x_i_ is the peak height of each drop-in observed, and n is the number of drop-in events [17, 45, 54]. The determined values of C and λ are shown in Table 1.

### 2.5. Stutter

In this study, only double-back/N-2 (B2), back/N-1 (B1), and forward/N+1 (F1) stutter models in STRmix were applied when assessing LRs. All the mixture profiles analyzed herein did not contain stutter peaks at the– 2bp position at SE33 and D1S1656. Stutter files that already exist within the software from a previously validated GF 29 cycle kit were used [36, 55]. EFM v2.1.0 models only back stutter [45, 54, 56]. Stutter types chosen to be modelled in STRmix (B1, F1, and B2) were retained in the input files after applying the AT values, and imported in both software even though F1 and B2 were not modelled in EFMv2.1.0. Any unmodelled stutter can also be explained as drop-in allelic events [39].

### 2.6. Variance parameters

Single source profiles (n = 333) obtained from the PROVEDIt database (filtered CSV files) were analyzed at an AT = 10 RFU at all the dye channels to maximize stutter observations of all the stutter types being modelled. A detailed description of the quality and quantity of the samples used in the calibration set is listed in S3 Table.

The α, β parameters describing the gamma distribution (Gamma (α, β)) of the allele peak height variance (*c*^2^) and stutter peak height variances (*k*^2^), and the mean of the locus-specific amplification efficiency variance (LSAE) derived from the Model Maker analysis (shown in Table 1) were set into the software prior to the interpretation of the DNA mixture profiles.

### 2.7. LR calculations and data analysis

The strength of evidence was assessed after setting parameters specific to each software as summarized in Table 1. STRmix interpretations were undertaken using the recommended MCMC parameters (shown in Table 1) [46]. In follow up analyses two interpretations were repeated with an increase in the number of accepts (1,000,000 burn-in and 500,000 post burn-in accepts per chain) to allow each of the chains to explore more possibilities in the probability space [59]. The reported sub-source LRs within the STRmix reports were considered for the analysis in this study.

LR calculations in EFM were performed using the maximum likelihood estimate (MLE) method with both the degradation and stutter statistical models jointly turned on and included in all the EFM analysis. The reported sub-source LRs within the EFM labeled as MLE based LRs were used in the data analysis.

The true NOC (ground truth) was specified in the settings of the software for each mixture profile that was interpreted. Each of the PROVEDIt mixture profile was compared to the appropriate known contributors (S4 Table) and known non-contributors (S5 Table). The known non-contributors were real (true-genotype) profiles randomly selected from the NIST 1036 US population dataset [57].

The allele frequencies and coancestry coefficient (*F*_ST_ or θ) set in both software for LR calculations are shown in Table 1. The propositions considered and the total number of propositions generated from each software are outlined in Table 2.

**Table 2:** Summary of the total number of PROVEDIt mixture profiles and H1-true and H2-true propositions analyzed in both STRmix and EFM for 2P, 3P, and 4P mixtures.

Number of contributors	Number of mixtures	Propositions	Number of H1-true tests	Number of H2-true tests
2P	154	H1: POI + U1	308	308
		H2: U1 + U2		
3P	147	H1: POI + U1 + U2	441	441
		H2: U1 + U2 + U3		
4P	127	H1: POI + U1 + U2 + U3	508	508
		H2: U1 + U2 + U3 + U4		

POI indicates the person of interest that can be either known contributor or known non-contributor. U1, U2, U3, and U4 indicate one, two, three, or four unknown, unrelated individual(s) to the mixtures. For each mixture, we performed as many known contributor LR analysis (H1-true tests) as there are contributors to each mixture. For each contributor analysis, a non-contributor LR analysis (i.e. single H2-true test) was also performed using real (true-genotype) profiles randomly chosen from NIST 1036 US population dataset [57].

All the LR values yielded from both software are reported in log_10_ scale in S4 Table (log_10_(LRs) for H1-true tests) and S5 Table (log_10_(LRs) for H2-true tests) with the corresponding combination of comparisons (mixture vs POI). The profile LRs and the per-locus LRs assigned by STRmix and EFM were for the 21 autosomal STR markers only. LR assessment for the gender and Y-STR markers, Amelogenin, Y-indel, and DYS391, were not considered by either software.

All data analysis and visualization discussed were conducted using the open source software **R** [60].

## 3. Results and discussion

### 3.1. Empirical assessment of LR systems using discrimination performance of H1-true and H2-true LR distributions

**Fig 2:**
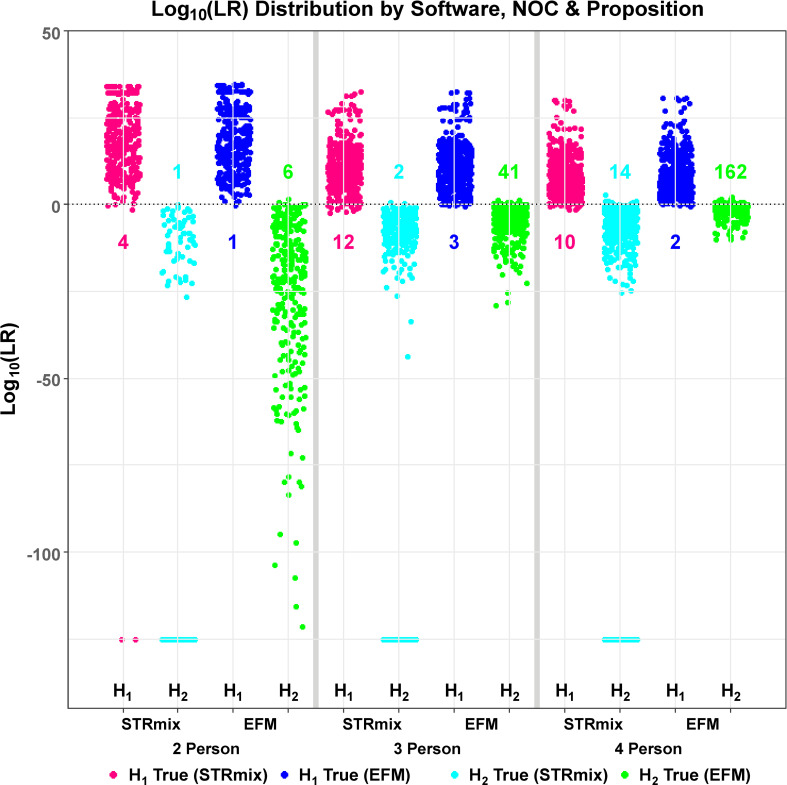
Distribution of log_10_ (LR) values for H1-true and H2-true tests assessed by STRmix and EFM for two, three, and four person mixtures. The x-axis shows the labels of propositions (H1 and H2), software (STRmix and EFM), and the NOC = 2 Person, 3 Person, and 4 Person. LRs are plotted on the y-axis as log_10_(LR) values. All samples from different mixture ratios, total DNA template amounts, and DNA treatments are built into this global/overall distribution plot. The plot contains a total of 308 H1-true tests and 308 H2-true tests for the 2P analysis, 441 H1-true and 441 H2-true calculations for the 3P analysis, and 508 H1-true and 508 H2-true tests for the 4P mixtures. STRmix provides an LR value of 0 for excluded loci resulting in profile LR of 0, while EFM gives a non-zero LR value (generally very close to zero). Profiles with LR results of 0 from STRmix are plotted at −125 on the log_10_ scale. ** Two H1-true test interpretations of 2P mixtures for which STRmix assigned profile LRs of 0 (plotted at H1 true STRmix NOC = 2 Person in magenta at −125 on the log_10_ scale and discussed in detail in Section 3.6).

We first examined the overall performance of the two systems to ensure that we are not comparing two PG models with substantially different discriminating performance. The distributions of the assigned log_10_(LR) values were plotted as function of NOC (2P, 3P, and 4P), propositions (H1 and H2), and software (STRmix and EFM) (Fig 2). The overall distribution plot shown in Fig 2 was further broken down by varying mixture ratios (S1 File) and different DNA treatments used to compromise the DNA quality of the samples (DNA damage, DNA degradation, and PCR inhibition) (S2 File).

The magenta and blue data points are the log_10_(LRs) of the H1-true tests generated in STRmix and EFM, respectively. Log_10_(LRs) of the H2-true tests assigned by STRmix and EFM are shown in cyan and green, respectively (Fig 2, and S1 and S2 Files). The distribution of log_10_(LRs) from the H1-true tests is well separated from the distribution of log_10_(LRs) from the H2-true tests when the quality and DNA template amount of the contributor or total template amount of the samples are sufficiently high and the NOC in a mixture profile is low. As the quality and template amount per contributor of interest or mixture profile decreases and/or the NOC increases, log_10_(LRs) assigned from H1-true tests and H2-true tests become less discriminatory and trend downwards and upwards towards 0 (horizontal line), respectively, (Fig 2, and S1 and S2 Files). Furthermore, as expected, when the distinction between the major-minor contributions to the same mixture increases so does the LRs of the major contributors as opposed to mixtures with equal contributor proportions (S1 File). As expected, the latter have lower LRs since information content associated with peak heights is limited or has no effect on LR calculations [3, 61–64].

**Fig 3:**
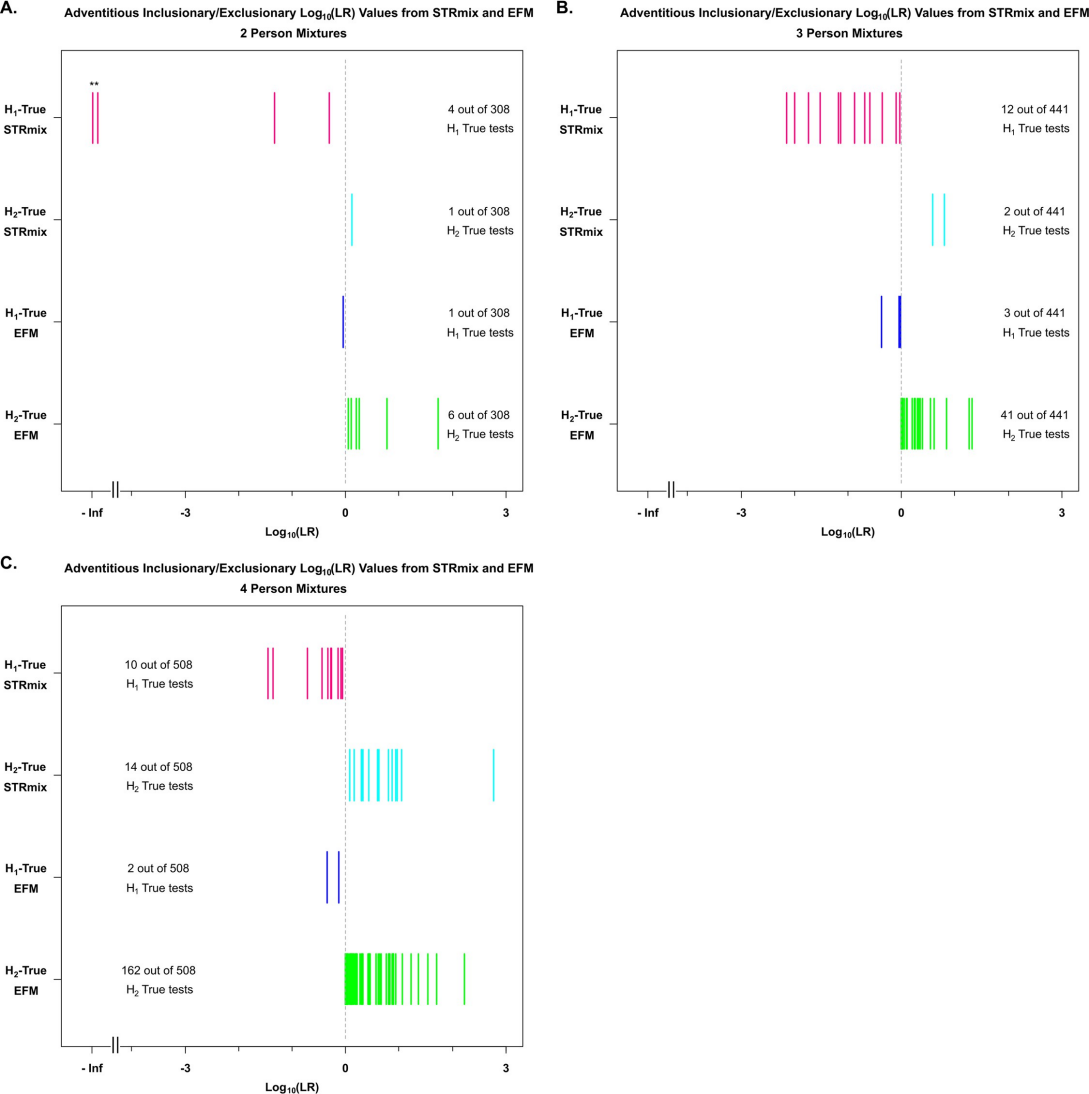
Summary of adventitious exclusionary and inclusionary support from both LR systems with their corresponding log_10_(LR) values. The x-axis shows the log_10_(LR) values for these adventitious exclusionary and inclusionary cases. The y-axis shows the labels of the tested propositions (H1 and H2) from each software (STRmix and EFM). ** in (A.) are the two 2P H1-true test interpretations for which STRmix assigned profile LRs of 0 (plotted in magenta at–Infinity (-Inf) on the log_10_ scale and discussed in detail in Section 3.6).

The magenta and blue data points below the central dashed horizontal line plotted at log_10_(LR) of zero in Fig 2 and S1 and S2 Files, correspond to the analyses of known contributors within STRmix and EFM that yielded log_10_(LRs) < 0 (adventitious exclusionary LRs). Cyan and green points above the horizontal line at log_10_(LR) = 0 in Fig 2 and S1 and S2 Files are instances of H2-true tests that yielded log_10_(LRs) > 0 (adventitious inclusionary LRs). The number of these adventitious inclusionary and exclusionary LR instances are indicated in Fig 2. These profiles are also presented with their corresponding log_10_(LRs) in Fig 3 and S6 and S7 Tables and are discussed in further details in Section 3.2.

Visual comparisons of the global aggregate of log_10_(LRs) in the distribution plot of Fig 2 indicate qualitatively that STRmix and EFM seem to have equal ability in discriminating between H1-true and H2-true scenarios. Both LR systems indicate better discrimination performance for lower complexity mixtures than for higher complexity mixtures (mixtures characterized by an increase in NOC and/or decrease in DNA quantity and quality). These qualitative observations are substantiated statistically in Section 3.3.

### 3.2. Overall specificity and sensitivity of the two LR systems

In this section we discuss overall specificity and sensitivity (Table 3) and instances of adventitious exclusionary LRs of which H1-true tests resulted in LR < 1 and cases of adventitious inclusionary LRs of which H2-true tests yielded LR > 1 across both NOC and LR systems (Fig 3 and S6 and S7 Tables).

**Table 3:** Summary of the number of observations and frequency (%) of known contributor analyses (H1-true tests) and known non-contributor analyses (H2-true tests) that yielded log_10_(LR) values > 0 (or LR > 1) and log_10_(LR) values < 0 (or LR <1), respectively.

H1-True Tests: LR > 1					H2-True Tests: LR < 1				
	STRmix		EFM			STRmix		EFM	
# of contributors	Counts	Frequency %	Counts	Frequency %	# of contributors	Counts	Frequency %	Counts	Frequency %
2 (N = 308)	304	98.70	307	99.68	2 (N = 308)	307	99.68	302	98.05
3 (N = 441)	429	97.28	438	99.32	3 (N = 441)	439	99.55	400	90.70
4 (N = 508)	498	98.03	506	99.61	4 (N = 508)	494	97.24	346	68.11
Total (N = 1,257)	1,231	97.93	1,251	99.52	Total (N = 1,257)	1,240	98.65	1,048	83.37

N represents the total number of either H1-true tests or H2-true tests conducted for the different number of contributors.

Across all the 2P, 3P, and 4P mixtures, 97.93% and 99.52% of H1-true test LRs assigned by STRmix and EFM, respectively, were greater than 1 (or log_10_(LR) > 0) (Table 3) while 98.65% and 83.37% of H2-true test LRs assigned in STRmix and EFM, respectively, resulted in LRs lower than 1 (or log_10_(LR) < 0) (Table 3). The number of observations and frequency values are broken down by NOC and LR systems as shown in Table 3.

#### 3.2.1. Adventitious exclusionary support (examples of LR < 1 when H1 is true)

There were instances of adventitious exclusionary LRs for true contributor analyses (H1-true tests) within both LR systems that returned log_10_(LRs) < 0 as illustrated in Fig 3 and S6 Table. Across the 1,257 H1-true tests conducted, there were 26 instances of adventitious exclusionary support with STRmix (4 out of 308 with 2P profiles, 12 out of 441 with 3P profiles, and 10 out of 508 with 4P profiles) and 6 instances with EFM (1 out of 308 with 2P profiles, 3 out of 441 with 3P profiles, and 2 out of 508 with 4P profiles) of which log_10_(LR) values for the POI were below 0. These are shown with their corresponding log_10_(LRs) in Fig 3 and S6 Table. As expected from the behavior of the LR [65] and as shown in S6 Table, all the cases of H1-true tests with log_10_(LRs) < 0 from both LR systems mainly occurred when comparing the minor contributors to DNA mixture profiles that contained limited amount of information due to low minor template amount (e.g. ≤ 63 pg), low total template amount, compromised/degraded DNA, loci with allelic dropout, increase in the number of contributors, stochastic variation causing confounding information from the allelic and stutter peaks, and allele sharing between contributors [7].

The number of instances of H1-true tests with log_10_(LRs) < 0 was greater with STRmix than EFM. However, the log_10_(LRs) generated in EFM for these STRmix cases were mostly true inclusions of low-level LR range between (1–1,453) (i.e., uninformative or slightly to moderately supporting H1 over H2) with the exception of three 2P instances that are discussed in Section 3.4. For example, as seen in S6 Table, when mixture F10_RD14-0003-39_40–1;2-M3c-0.045GF was compared to the minor contributor “39”, STRmix gave a log_10_(LR) of -0.2 while EFM gave a log_10_(LR) of 0.9.

#### 3.2.2. Adventitious inclusionary support (examples of LR > 1 when H2 is true)

There were also instances of adventitious inclusionary LRs for known non-contributor analyses (H2-true tests) that returned log_10_(LRs) > 0 within both LR systems as illustrated in Fig 3 and S7 Table. Out of the 1,257 total H2-true tests performed for 2P, 3P, and 4P, there were 17 log_10_(LRs) greater than zero analyzed with STRmix (1 out of 308 with 2P, 2 out of 441 with 3P profiles, and 14 out of 508 with 4P profiles) and 209 log_10_(LRs) greater than zero with EFM (6 out of 308 with 2P profiles, 41 out of 441 with 3P profiles, and 162 out of 508 with 4P profiles). These cases are presented with their corresponding log_10_(LRs) in Fig 3 and S7 Table. The largest observed LR for the known non-contributors assigned by STRmix was 587 (log_10_(LR) = 2.7) and in EFM was 167 (log_10_(LR) = 2.2), when comparing a known non-contributor with the 4P mixture D02_RD14-0003-40_41_42_43–1;1;1;1-M2e-0.124GF.

As expected, positive log_10_(LRs) obtained from non-donors in both software were attributed to one or more of the following: increased complexity of mixtures, increase in the number of contributors, mixtures generated from low total template and/or compromised low quality DNA, stochastic effects, and chances of allele sharing between the non-contributor profiles and evidence profiles [2, 65–67].

The number of instances of positive log_10_(LRs) from non-contributors were greater with EFM than STRmix (Fig 3 and S7 Table). The LR values assigned by EFM were based on the MLE method, an approach that has elevated rates of LR > 1 for the H2-true tests than the conservative method as stated and observed in [54, 62]. However, these adventitious inclusionary LRs were low-level with range of values between 1 to 53 (i.e., uninformative or slightly supporting H1 over H2) (S7 Table) [54, 62, 68].

### 3.3. Using empirical Receiver Operating Characteristic (ROC) plots to study discrimination performance of the LR systems

**Fig 4:**
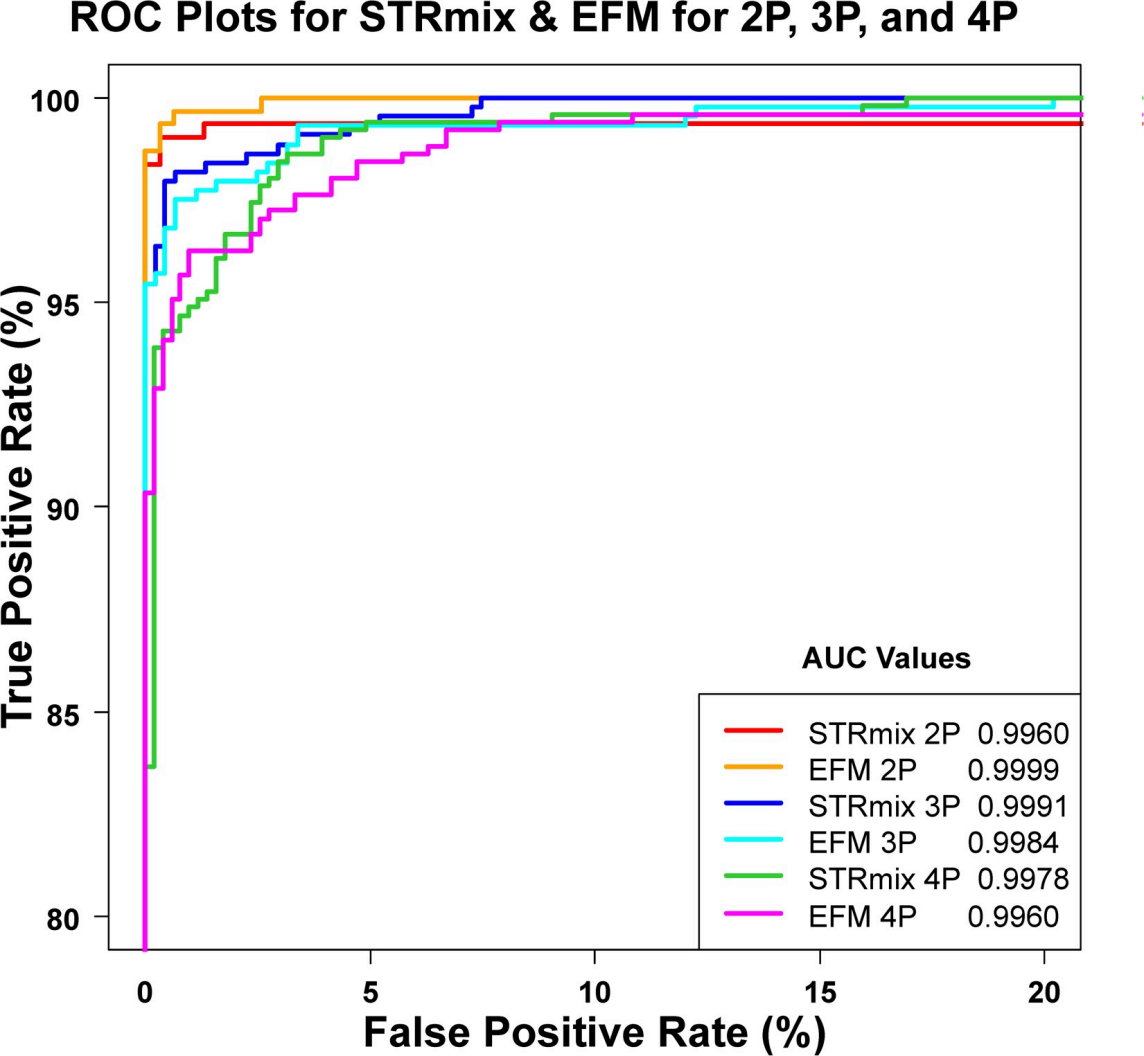
Empirical ROC plots used to study discrimination performance of the LR systems. ROC plots are built per varying NOC and software. Each NOC dataset is composed of profiles of different DNA quality, quantity, and mixture proportions. The red, blue, and green curves are the ROC plots constructed using LR values of known contributors and known non-contributors of 2P, 3P, and 4P mixtures analyzed within STRmix, respectively. ROC plots constructed with LR values assigned by EFM are shown in orange (2P), cyan (3P), and magenta (4P). The plot contains a total of 308 H1-true tests and 308 H2-true tests for the 2P analysis, 441 H1-true and 441 H2-true calculations for the 3P analysis, and 508 H1-true and 508 H2-true tests for the 4P mixtures. The calculated AUCs and p-values are shown. All p-values were > 0.05.

We used Empirical Receiver Operating Characteristic (ROC) plots [69] as statistical tools to quantify the discrimination performance between the H1-true scenarios and H2-true scenarios of the two different LR systems. The discrimination performances were quantified using a numerical metric, the Area Under ROC Curve (AUC). AUC is the area between each ROC plot and the horizonal x-axis (Fig 4). Statistical tests (p-values) for AUC comparisons (i.e., differences between the ROC plots) were calculated and listed in Fig 4 [70].

**Table:** 

Comparison Group	*P-values*
STRmix 2P *vs* EFM 2P	0.1638
STRmix 3P *vs* EFM 3P	0.1093
STRmix 4P *vs* EFM 4P	0.1859

LR values of the H1-true tests and H2-true tests were combined across each NOC level (2P, 3P, and 4P) generated from each software (STRmix and EFM), thus creating six datasets: STRmix 2P, EFM 2P, STRmix 3P, EFM 3P, STRmix 4P, and EFM 4P. To construct the ROCs shown in Fig 4, a series of various LR thresholds were applied to each of the 6 datasets generating true positive rates (TPR) and the corresponding false positive rates (FPR). TPR represented the counts of the true contributors of which LR values were > a given threshold value divided by the total counts of the known contributors in the considered dataset. FPR represented the counts of the known non-contributors with LR values > a given threshold value divided by the total counts of known non-contributors in the considered dataset. ROC plots were created by plotting the TPR (along vertical axis) versus the FPR (along horizontal axis). The p-values of the comparisons of areas under the ROC plots of: STRmix 2P vs EFM 2P, STRmix 3P vs EFM 3P, and STRmix 4P vs EFM 4P were > 0.05 (Fig 4), indicating that for the considered data the differences between the two software in the ability to discriminate between H1-true and H2-true scenarios were not statistically significant.

**Fig 5:**
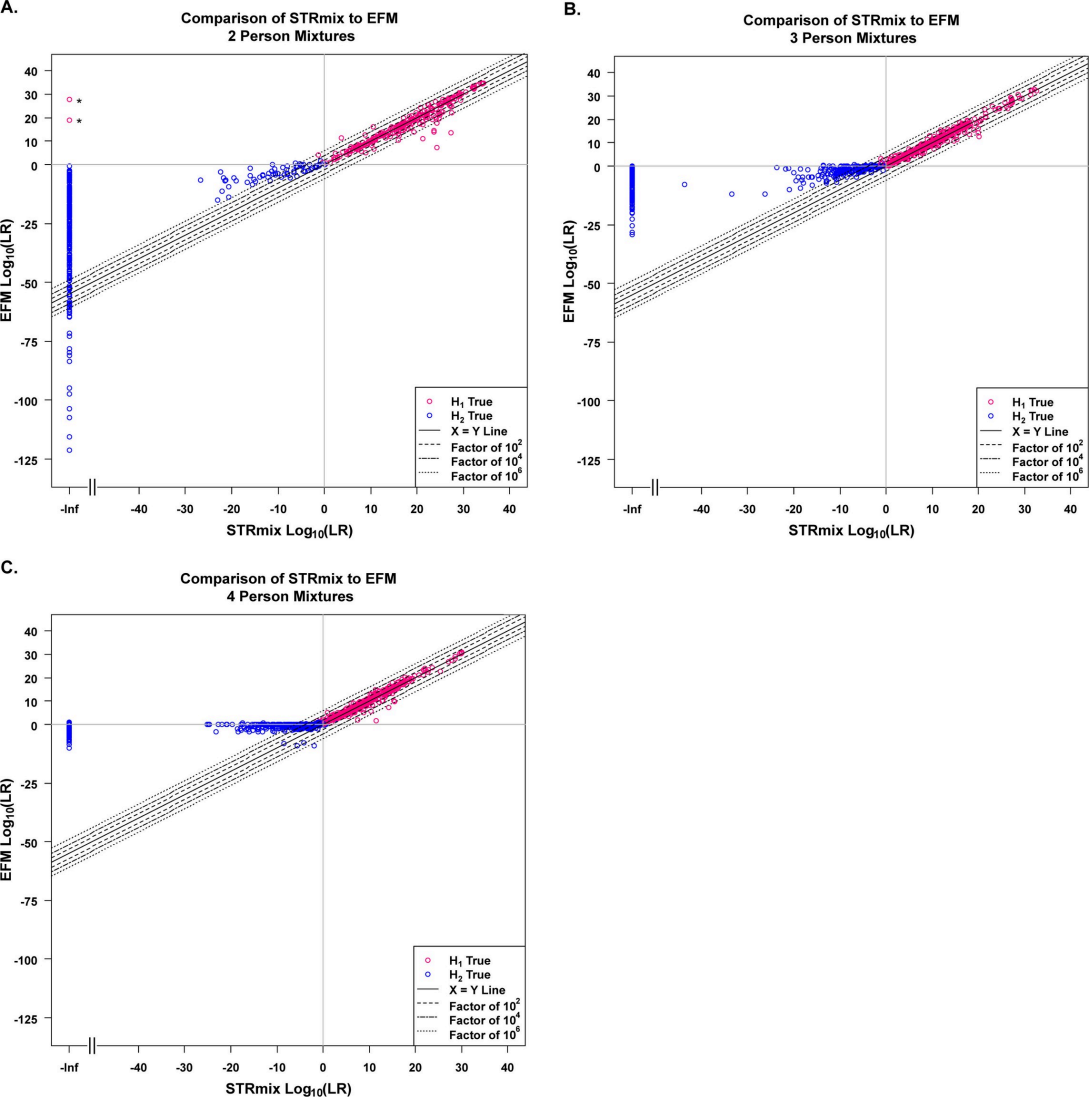
Global overall profile H1-true test and H2-true test log_10_(LR) values assigned by STRmix and EFM. ** in (A.) are the two 2P H1-true test interpretations for which STRmix assigned profile LRs of 0 (plotted in magenta at–Infinity (-Inf) on the log_10_ scale and discussed in detail in Section 3.6).

The ROC plots shown in Fig 4 statistically support the qualitative observation visualized in the distribution plots of Fig 2. Therefore, the ability for the two LR systems to discriminate between known contributors and known non-contributors are statistically indistinguishable for the data considered. However, that does not imply that STRmix and EFM are producing equal LR values or agreeing when the same profile is being interpreted within both software. Sample to sample comparisons are discussed in Section 3.4. Rather the plots in Figs 3 and 5 are considering the data in aggregate.

### 3.4. Global overall profile log_10_(LR) values of H1-true tests and H2-true tests from each LR system

Scatter plots (Fig 5) were produced by plotting the log_10_(LRs) of the H1-true tests (magenta datapoints) and the H2-true tests (blue datapoints) obtained from STRmix on the x-axis against the corresponding log_10_(LRs) assigned using EFM on the y-axis for the 2P (Fig 5A), 3P (Fig 5B), and 4P (Fig 5C) mixture profiles. Identical or near identical log_10_(LR) values assigned by both LR systems fell on the solid black 45° degree line, X = Y. Datapoints that did not fall on the diagonal line corresponded to instances with varying degrees of difference in the overall LR profile between the two LR systems. For example, datapoints located within the two black dashed lines, two black dash-dotted lines, and two black dotted lines surrounding the line X = Y, corresponded to cases with LR results differing by a factor as high as 10^2^, 10^4^, and 10^6^, respectively (Fig 5). Datapoints that are outside the pair of black dotted bands represented LRs assigned by the two software that differed by more than a factor of 10^6^. These differences represented instances where either the LRs obtained from STRmix exceeded the ones obtained in EFM or vice versa. It is interesting to note that differences in the assigned LR values were greater with the non-contributor testing profiles than with the H1-true testing cases. Instances that differed by factor of ≥ 10^3^ and the potential explanations for the differences will be discussed in Section 3.6. Impacts of the differences in the inter-software numerical LRs on verbal expression will be discussed in Section 3.7.

To conclude this section, although both LR systems show comparable discrimination performance, differences exist in log_10_(LR) values on a case-by-case basis. Differences in log_10_(LR) values assigned by STRmix and EFM at the profile level covered a wide range from zero to over a million (discussed in detail in Sections 3.5 and 3.6) for the same input data (i.e., the same EPG). The differences appear to be greater in the H2-true cases than in the H1-true cases.

### 3.5. Distribution of differences in log_10_(LR) values between the two LR systems

**Fig 6:**
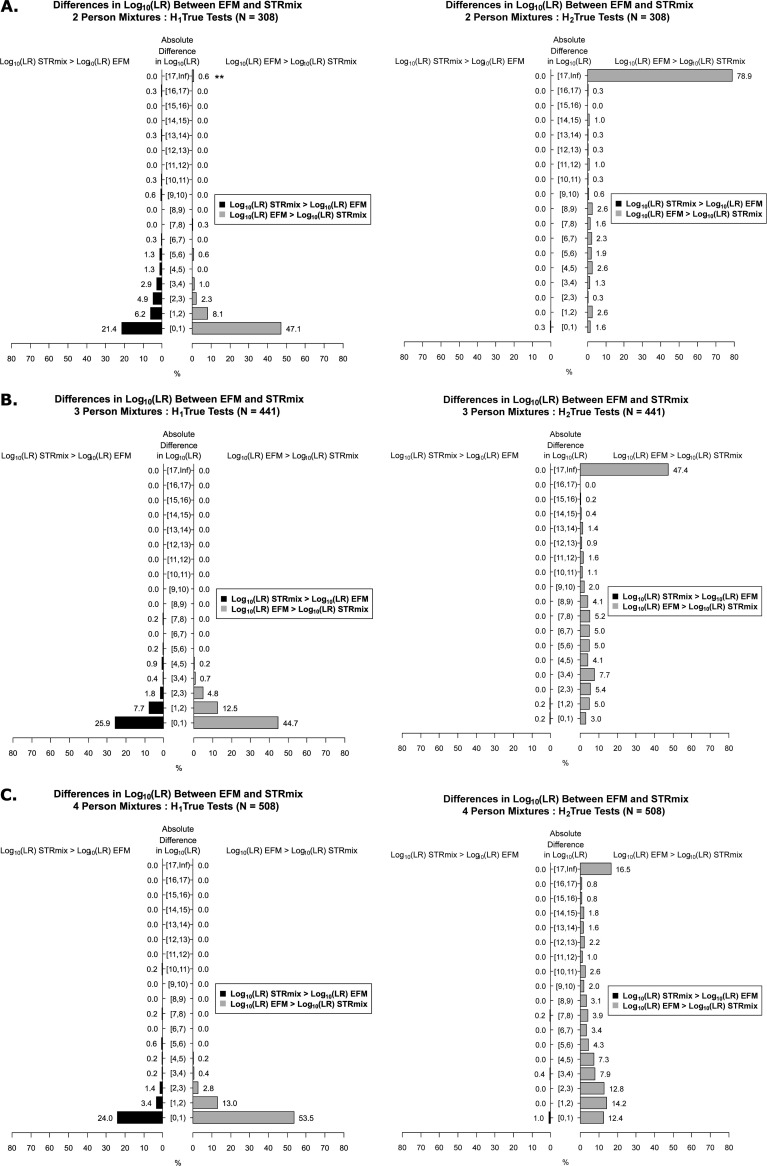
Relative frequency histograms of the degree of differences in log_10_(LR) values between the two LR systems. The absolute difference in log_10_(LR) are shown on the y-axis. The square bracket “[" in the interval notation "[)" indicates that the endpoint is included in the interval and the parenthesis ")" in the interval notation "[)" indicates that the endpoint is not included. For example, [1, 2], is the interval of values between 1 and 2, including 1 and up to but not including 2, i.e., 1 ≤ values < 2. The x-axis shows the relative frequencies (in %) of the differences in log_10_(LR) values between the LR systems occurring within each bin. The relative frequencies are also labeled above each bar of the histogram. ** are the two 2P H1-true test interpretations for which STRmix assigned profile LRs of 0 (binned into the [17, Inf) category and discussed in detail in Section 3.6).

**Fig 7:**
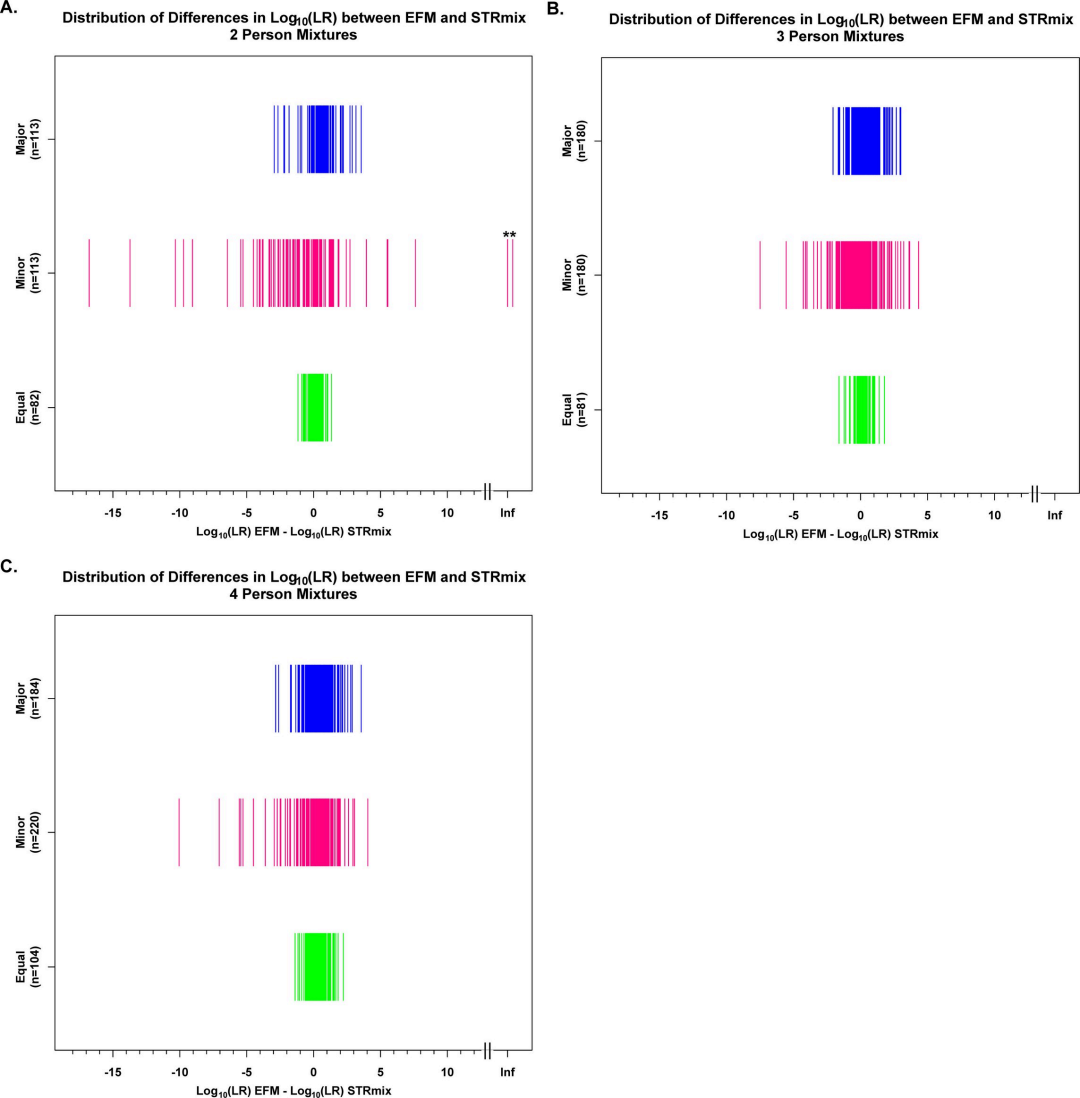
Distribution of differences in log_10_(LRs) across major, minor, and equal contributors. The differences in log_10_(LRs) here shown between EFM and STRmix (log_10_(LR)_EFM_−log_10_(LR)_STRmix_) are plotted on the x-axis in log_10_ scale. The y-axis shows the labels of the types of POI with their corresponding number of observations. ** are the two 2P H1-true test interpretations for which STRmix assigned profile LRs of 0 (plotted in magenta at Infinity (Inf) on the log_10_ scale and discussed in detail in Section 3.6).

Here, we describe and plot the degree and distribution of the observed differences between the two LR systems. The actual differences in log_10_(LRs) were calculated in both directions (i.e., log_10_(LR)_STRmix_−log_10_(LR)_EFM_ as well as log_10_(LR)_EFM_−log_10_(LR)_STRmix_) for the H1-true tests and H2-true tests (histograms shown in Fig 6). These differences were broken down into factor of 10 bins for the 2P (Fig 6A), 3P (Fig 6B), and 4P (Fig 6C) analysis and the relative frequencies (in %) of these differences are indicated for each bin in Fig 6. For example in Fig 7A, 21.4% and 47.1% of the differences for the 2P H1-true tests were between 0 to 1 on log_10_ scale for log_10_(LR)_STRmix_—log_10_(LR)_EFM_ (black histograms) and log_10_(LR)_EFM_—log_10_(LR)_STRmix_ (grey histograms), respectively. The relative frequency histograms (Fig 6) indicate that (i) the differences between the two LR systems were smaller with the H1-true testing cases than with the non-contributor tests and (ii) EFM tended to give higher LR values than STRmix for both the H1-true tests and H2-true tests.

The actual differences in log_10_(LRs) for the H1-true tests were further stratified by the type of POI (i.e., major, minor, and equal contributors as defined in S4 Table) constituting the 2P (Fig 7A), 3P (Fig 7B), and 4P (Fig 7C) mixture profiles. As shown from the distribution plots in Fig 7, the magnitude of the differences for the two LR systems were greater for the minor contributors (shown in magenta) than for the major (shown in blue) and for the equal (shown in green) contributors. LRs assigned by STRmix and EFM agreed more when POI(s) constitute the equal contributors of the mixture (Fig 7). This is expected because with balanced profiles, peak height information content has less effect on LR calculations than in cases of major: minor profiles [3, 61–64].

### 3.6. Evaluation of apparent differences in log_10_(LR) values between the two LR systems

In this section we discuss the steps performed to further investigate differences in the assigned LR values obtained from the two LR systems on a case-by-case basis, where the differences are observed, and the potential explanations for these differences. We restrict our discussion to instances when LR (STRmix) ≥ 1000*LR (EFM) that constituted 7.3% of the 2P, 1.7% of the 3P, and 1.4% of the 4P H1-true tests (histograms of Fig 6 and S11 Table) and instances when an LR (EFM) ≥ 1000*LR (STRmix) that accounted for 2.5% of the 2P, 0.9% of the 3P, and 0.6% of the 4P H1-true tests (histograms of Fig 6 and S12 Table). Only differences in H1-true results (true known contributor samples) are discussed.

LR computations obtained from the two software were based on same/fixed EPG features, same pair of propositions, NOC, theta, and population allele frequency. Therefore, results presented here shows that differences observed in LR values can occur due to one or more of the following reasons:

- Nonconvergence of the Markov Chain Monte Carlo (MCMC) algorithms and MLE
- Decision to provide identical EPGs for both LR systems
- Different modeling assumptions and parameters settings between the two software

We discuss each of the above reasons and provide examples from the data set. The availability of both the mixture and reference profiles was beneficial and helped in the investigation of observed differences of the assigned LR values.

#### 3.6.1. Non-convergence of the MCMC algorithms and MLE

STRmix pdf reports contain summary statistics for each interpretation conducted in the software and can be used by analysts as diagnostics on the performance of the interpretation according to the specified models. These diagnostics have been classified into primary and secondary categories and are discussed in detail in Russell et al. [59]. In actual casework, every analysis should be subjected to diagnostic checks. But in this study and for practical reasons only cases where STRmix and EFM differed by a factor of ≥ 10^3^ were inspected for genotypic weights, mixture proportions, per-locus LRs, log(likelihood), peak height variance parameters, and Gelman-Rubin (GR) statistics.

**Fig 8:**
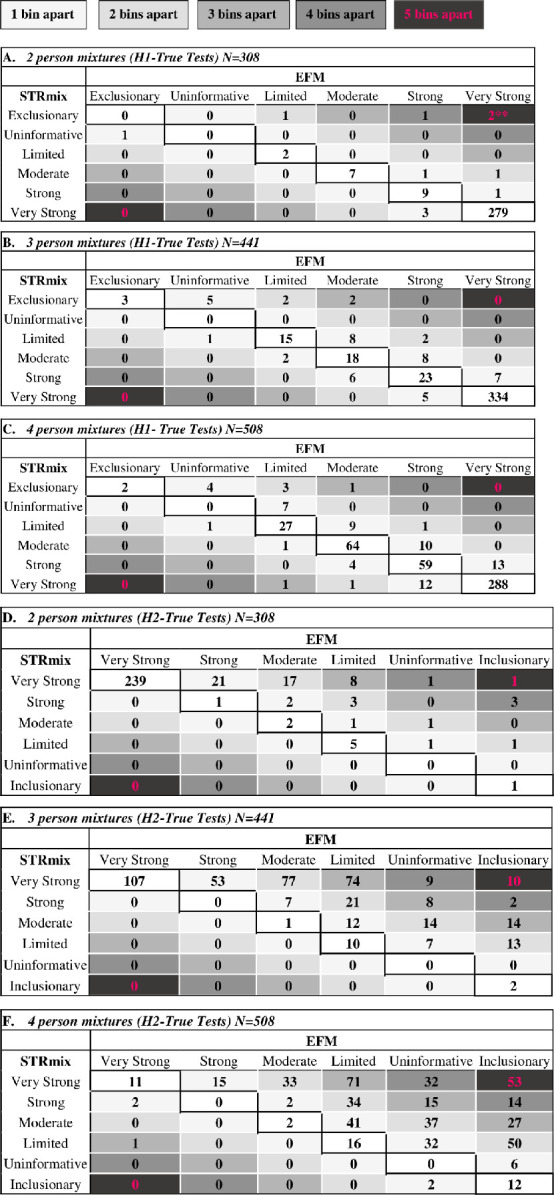
Concordance/discordance tables of the binned LR values assigned by STRmix and EFM into their verbal equivalents. The tables display the results of the categorization of the LRs for both the H1-true tests of (A) 2P where ** are the two 2P H1-true test interpretations for which STRmix assigned profile LRs of 0 (binned into the Exclusionary category and discussed in detail in Section 3.6), (B) 3P, and (C) 4P and H2-true tests of (D) 2P, (E) 3P, and (F) 4P generated in STRmix and EFM into their corresponding verbal expression. Also, the tables demonstrate the observed differences in the verbal expressions between the two LR systems. The number of cases that resulted in same verbal expression between STRmix and EFM fell inside the diagonal (white cells). All the numbers outside the diagonal (shaded cells) are indication of cases where LRs from both software were classified into different categories and resulted in shifting by one or more than one verbal category (indicated by different shades as shown by the legend). Values in and above the diagonal are the results of the verbal expression of LRs produced in EFM while values in and below the diagonals are the results of the verbal expression of LR values assigned by STRmix. The verbal expressions are shown at the top and left edges of the tables.

Two extreme differences observed between STRmix and EFM were with the 2P mixture profiles, C02_RD14-0003-40_41–1;4-M2U15-0.315GF (herein referred to as “C02”) and H06_RD14-0003-48_49–1;4-M2e-0.315GF (referred to as “H06”) (S8 Table). C02 and H06 generated profile LR of 0 in STRmix when compared to true known minor contributors, 40 and 48, respectively. A locus LR value of 0 will lead to a profile LR of 0. The log_10_(LR) assessments for these profiles in EFM were 27.6 for C02 and 19.0 for H06. Unlike STRmix, EFM displays low to very low LRs for exclusionary loci but does not provide a zero locus LR. A review of the per locus LRs (S8 Table) assigned to the evaluation of the POIs in STRmix indicated that almost all loci favor inclusion (LR > 1) except for a single locus displaying an LR of 0 in each interpretation, D1S1656 in C02 and D3S1358 in H06. Instances of single locus LR = 0 have been observed using different data from different studies [2, 36, 71]. In such cases and if samples are sufficient, either replicate analysis or sample reamplification is used. Otherwise, options are to either ignore that locus during deconvolution, or repeat the deconvolution in STRmix with: a random starting seed for the MCMC different than the one that gave LR = 0, or an increase in number of MCMC accepts, or a larger Random Walk Standard Deviation (RWSD) [2, 7, 36, 59, 71]. Here, we repeated the runs in STRmix with more MCMC accepts (as discussed in Section 2.7) and the repeated interpretations generated non-zero LRs for the affected loci, and profile log_10_(LRs) of 24.8 and 19.6 (S8 Table). It is to note that these two discussed 2P H1-true test interpretations with profile LRs of 0 assigned by STRmix were plotted: (i) at −125 on the log_10_ scale in Fig 2 and S1 and S2 Files; (ii) at–Infinity (-Inf) in Figs 4A and 6A; (iii) at Infinity (Inf) in Figs 7A and 8A; and were binned into the exclusionary verbal category (Table A in Fig 8).

Another extreme difference observed between STRmix and EFM was with E04_RD14-0003-42_43–1;9-M2U105-0.15GF, a 2P mixture profile of which comparison to the minor contributor in STRmix and EFM, yielded log_10_(LRs) of -1.3 and 4.1, respectively (EPG and data shown in S4 Table). A review of the STRmix output indicated negative log(likelihood), which might be due to several reasons including “flawed input data” [2, 59]. Inspection of the DNA typing results, ground truth genotypes of the POIs, and deconvolution results indicated retained artifact peaks binned into alleles at two loci, D19S433 “18.2” and D5S818 “14” (EPG shown in S9 Table). The artifacts were each modelled in STRmix as being allelic in origin and were included in the genotypic combinations thus leading to exclusion after comparing the resolved profile to the true contributors [7, 71]. In such cases, mixture samples can be re-injected or reamplified [2]. However, since only the electronic data was accessible for this study, the artifacts were removed and the input file were re-interpreted in both STRmix and EFM, generating profile log_10_(LRs) of 0.8 and 4.6, respectively (S9 Table).

A GR > 1.2 might be an indication that more MCMC runs may be needed for convergence [7, 59, 72]. Profiles indicating discrepancies of ≥ 10^3^ and with a GR > 1.2 (a total of 6 out of 53) were reinterpreted in STRmix using higher number of burn-in and post burn-in accepts [7]. The repeated LR computations resulted in lower GR. Although the GR decreased, there was either no effect or a slight increase by a factor of 10 in the profile overall LRs (S10 Table) and did not substantially alter the observed LR differences in these cases.

EFM provides an option for selection of one of four models (turning on either or both of the degradation and stutter models) under H1 and H2 hypothesis and generates a Probability-Probability (PP) plot to examine if the model selected explain the observed data adequately [56, 68, 73]. A linear trend of PP plots within 99% Bonferroni band indicates that the assumed continuous models may be adequate for the data of the observed peak heights above the detection threshold [56, 73]. Herein, we selected the model with both degradation and back-stutter options turned on and cross checked a total of four mixture profiles out of 53 interpretations showing discrepancies (S3 File). The PP plots showed that models selected (i.e., degradation ON and stutter ON) appear to adequately explain the data.

These observations indicate that non-convergence of MCMC and the inability of the software to describe the observed profile given the provided information are one of the reasons behind the observed differences.

II. Decision to provide identical EPGs for both LR systems

Instances of the underestimation of LR values observed in EFM as compared to STRmix was primarily due to the unmodelled stutter type peaks not filtered from the input files (S11 Table). Stutter models for B2, B1, and F1 were applied to the mixture deconvolutions performed in STRmix. EFM v2.1.0 used in this work only models stutter peak heights in the -1 repeat unit position [54, 56, 73]. The B2 and F1 peaks retained after applying the analytical thresholds and not pre-filtered from the DNA profiles before analysis in EFMv2.1.0 led to instances of smaller LRs than those assigned by STRmix (S11 Table). As reflected in (S11 Table), differences were highest with minor contributors in major/minor mixture profiles where allele peak heights from a minor contributor can have the same size and height as stutter peaks of major contributors [54, 59].

To further examine this hypothesis, we removed the retained (i.e., above AT) unmodelled F1 and B2 stutter peaks from the profiles that showed a difference of factor of ≥ 10^3^ and reinterpreted the analysis in EFM v2.1.0 for the 2P, 3P, and 4P mixtures. The LRs of the minor contributors in the repeated profiles increased substantially, thus decreasing the differences in log_10_(LR) values observed between STRmix and original EFM runs (S11 Table).

Our intentions of leaving in the unmodelled stutters (F1 and B2) were to have identical EPGs as input files for both software especially since according to certain publications any unmodelled stutter could be explained as drop-in allelic events [39, 54]. For example, according to You and Balding [39], “All the alleles explained by the over-stutter (OS) or double-stutter (DS) models could also be explained by the drop-in model, and so it is unclear whether or not there is a material benefit from modelling DS and OS in addition to drop-in, an option that is available in likeLTD”. According to Bleka et al. on the effect of applying the drop-in model to accommodate an extra allele in [54]: “Hence we observe that the implemented drop-in model in EuroForMix accommodates spurious alleles very efficiently—there is a small decrease in the LR. As expected, the larger the peak height, the greater the reduction in LR, because it impacts on heterozygote balance with other alleles.” These unmodelled stutter peaks were considered in certain cases less likely to be drop-ins than alleles and therefore were considered alleles instead as observed from the profile LRs, per locus LRs, and deconvolution. A new EFM version 3.2.0 [17, 74] is now available and accounts for forward stutter in LR calculations. This new version was not available during the time of the analysis.

Unmodelled stutter peaks (F1 and B2) can be removed before interpretation to improve the fit of the model to the observed data by using stutter-type specific thresholds [68]. However, there is no guarantee that the stutter thresholds will work all the time across all the cases due to false positives (stutter peaks are left in as alleles) and/or false negatives (removing low-level alleles of the minor contributors).

We discuss an illustration in S4 File on one of the profiles shown in S11 Table. D05_RD14-0003-48_49–1;4-M3a-0.315GF is a two-person mixed GlobalFiler (GF) DNA profile with major and minor contributors from the PROVEDIt dataset with pristine DNA (a) of total DNA amount of 315pg and mixture ratio of 1:4. When the POI corresponded to the major contributor, STRmix and EFM gave near identical profile log_10_(LR) values of 27.6 and 27.9, respectively. However, for the minor contributor position, STRmix and EFM gave profile log_10_(LR) values of 27.4 and 21.9, respectively, leading to a 5.4 difference in log_10_ scale (S4 File). A further review of the per-Locus LR tables obtained from STRmix and EFM for the minor contributor indicated that all loci had LR values favoring inclusion (i.e., LR > 1), except for the D22S1045 in EFM that has been assigned a locus LR < 1 (i.e., 0.001139) (S4 File). A review of the mixture profile (S4 File) indicated that the exclusionary LR at D22S1045 generated from EFM is likely due to a peak at “16” at D22S1045 which is likely an F1 of allele “15”. EFMv2.1.0 did not model F1 and had accounted for “16” as being allelic in origin instead of being modeled as “drop-in” (S4 File). We removed the “16” from the input file and reinterpreted in EFMv2.1.0. The rerun gave a D22S1045 locus LR of 16.2 (S4 File) and a profile log_10_(LR) of 26.1 (S4 File), thus decreasing the discrepancy between EFM and STRmix to a factor of approximately 10.

There were cases (e.g. A03-40_41–1;4-M2U105-0.315GF; H03-48_49_50_29–1;4;4;4-M3I22-0.75GF; E03-48_49_50_29–1;4;4;4-M2I15-0.75GF; D01-50_29_30_31–1;1;2;1-M2a-0.155GF), that did not contain any instances of F1 or B2 and differed by a factor of ≥ 10^3^ when compared to the profile LR generated in EFM (highlighted in red in S11 Table). A plausible explanation for these differences will be discussed below.

III. Different modeling assumptions and parameters settings between the two software

There were instances in which EFM assigned larger LR values than STRmix (S12 Table) and cases of which STRmix profile LRs were greater than EFM LRs (highlighted in red in S11 Table and as mentioned above not due to F1 or B2). Some of these profiles in which EFM assigned larger LR values than STRmix contained instances of F1 and B2. Reinterpreting those profiles in EFMv2.1.0 with F1 and B2 removed resulted in a slight increase or had no effect on the profile LRs (S12 Table). Larger differences between the two LR systems were observed when comparing minor contributors (in most cases) with mixture profiles composed of low total template amount, low minor template amount, and/or degraded DNA (as reflected in S12 Table). In these cases, there is increase in stochastic effects, variation in peak heights, and drop-out events.

As an illustration we discuss one of the profiles shown in S12 Table. B07_RD14-0003-48_49–1;4-M3e-0.075GF is a two-person mixed GlobalFiler (GF) DNA profile with major and minor contributors from the PROVEDIt dataset with degraded DNA (DNA treated with DNase I) of total DNA amount of 75 pg, minor template amount of 15 pg, and mixture ratio of 1:4. For the minor contributor, EFM and STRmix gave profile log_10_(LR) values of 11.3 and 3.6, respectively, leading to a 7.6 difference in log_10_ scale (S5 File). A further review of the per-Locus LR tables obtained from EFM and STRmix for the minor contributor indicated that the LR of D1S1656 had the largest difference (S5 File). The known genotypes at this locus for major and minor contributors were (12,15) and (13,14), respectively, showing that allele “13” dropped-out. A review of the STRmix deconvolution indicated that the genotype at that locus (Q,14) is accepted with a low assigned weight (S5 File). The weights in STRmix are used for LR assignments [59], hence the low D1S1656 LR value.

Therefore, differences observed in profile LRs between the STRmix and EFM maybe partly influenced by the analyst’s review of data and analyst’s decisions when interpreting DNA typing results, different modeling assumptions and statistical models between the two software (e.g. degradation’s effect on peak height, peak height variability, heterozygote balance, drop-in/drop-out, and different stutter types), parameter values settings, and how each software is implementing deconvolution and LR calculations [67]. Different analysts may make different decisions when interpreting the same EPG, thus leading to different LRs even if using the same software [37]. Upon changing models (e.g. modeling double-back and forward stutter) and/or changing parameter values (e.g. adding a per-dye detection thresholds in EFM v3.2.0, parameters from model maker and profiling kit in STRmix generated from internal validation studies) the resulting LRs will vary to some degree. Different algorithms will also lead to different deconvolution and LR values for the same DNA profile; EFM uses maximum likelihood approaches and STRmix uses Bayesian or MCMC approaches [56].

### 3.7. The verbal equivalents resulting from the numeric LR values from STRmix and EFM

The numeric LR values can be accompanied by a verbal expression, a qualitative statement used in court to describe the degree of support of the findings for one of the propositions relative to the alternative proposition [75–77]. As an exercise for this study we assessed if differences in the quantitative LRs assigned by the two different LR systems resulted in the same or different verbal expressions for both the H1-true tests and H2-true tests. The LR values assigned by STRmix and EFM were binned into their corresponding verbal categories based on the verbal convention recommendations set by the Scientific Working Group on DNA Analysis Methods (SWGDAM) [78] (shown in S13 Table). The SWGDAM verbal scale is composed of 5 verbal categories: ‘uninformative’, ‘limited’, ‘moderate’, ‘strong’, and ‘very strong’ for both H1 and H2 support. Each category is associated with a bracket of numerical range of LR values as shown in S8 Table.

For the H1-true tests (Tables A, B, and C in Fig 8 and S14 Table), the changes in the verbal statements increased with an increase in the number of contributors. The following analysis were binned into identical verbal categories: 96.42% (297 out of 308) of the LRs from 2P mixtures, 89.11% (393 out of 441) of the LR values of 3P mixtures, and 86.61% (440 out of 508) of the LRs of the 4P mixtures. Hence, (11 out of 308) of the LRs of 2P samples, (48 out of 441) of the LRs of 3P samples, and (68 out of 508) of the LRs of 4P samples were classified into different categories (Tables A, B, and C in Fig 8). For the 11 2P cases that were different verbally, 6 were placed in the neighboring categories (for example, for the same 2P profile, an LR from one software was binned into ‘moderate support’ and the LR from the other software was placed in the ‘strong support’ category). The other 5 cases were located in non-adjacent categories and differed by two or more than two verbal categories (e.g. ‘moderate support’ and ‘very strong support’ or ‘exclusionary’ and ‘limited’ or ‘exclusionary’ and ‘strong support’ or ‘exclusionary’ and ‘very strong support’) (Table A in Fig 8). With 3P analysis, (6 cases out of 48) were classified into non-adjacent categories: 4 cases were two categories away (‘exclusion’ and ‘limited support’ or ‘limited support’ and ‘strong support’) and 2 cases were different by three categories (‘exclusion’ and ‘moderate support’) (Table B in Fig 8). For the LRs of the 4P (Table C in Fig 8) analysis that fell in different categories, only 7 out of 68 cases were different by more than one verbal category: 5 cases were different by two categories (“Exclusion” and “Limited Support” or “Limited Support” and “Strong Support” or “Very Strong Support” and “Moderate Support”), and 2 cases were three categories away: (“Exclusion” and “Moderate Support” or “Very Strong Support” and “Limited Support”). Cases of LRs with more than one category difference corresponded to H1-true tests in which POI was a minor contributor and/or had low template amount (S14 Table).

The categories used for the binning the LRs of the H2-true tests are in favor of H2 over H1 (i.e., mirror image of the verbal scale of the H1-true tests). For the H2-true tests, similarly as for the H1-true tests, as the number of contributors increased the differences in the verbal statements increased as well (Tables D, E, and F in Fig 8 and S15 Table). The following analysis were binned into the same verbal category: 80.51% (248 out of 308) of the LRs from 2P mixtures, 27.21% (120 out of 441) of the LR values of 3P mixtures, and 8.07% (41 out of 508) of the LRs of the 4P mixtures (Tables D, E, and F in Fig 8). For the 60 2P cases that were different verbally, 35 were placed in non-neighboring categories (Table D in Fig 8). With 3P and 4P analysis, (242 cases out of 321) and (367 cases out of 467), respectively, were classified into non-adjacent categories (Tables E and F in Fig 8).

## 4. Conclusion

In this independent study, we examined the discrimination performance as well as LR values assigned by two LR systems using two continuous PGS built on different modelling assumptions, STRmix (proprietary) and EFM (open-source) [7, 56]. We use the term LR system deliberately to emphasize that the assigned LR values are a product of the decisions that went into the interpretation process of the LR system and not solely the PGS. For example, our specific choice of the PROVEDIt filtered files, protocols used for the data analysis in both STRmix and EFM, decision to use the known NOC, and to provide similar data (EPGs) into both software are specific to “our” LR system used in this study. We recognize that alternative decisions could have been made, and thus different LR values could have been assigned. We described the degree of differences in the LR values, where the differences occur, and the potential explanations for the observed differences. We analyzed 154 2P, 147 3P, and 127 4P mixture profiles from PROVEDIt database [43, 44] of varying DNA quality, DNA quantity, and mixture ratios (shown in S4 and S5 Tables). Both H1-true tests (S4 Table) and H2-true tests (S5 Table) for the 2P, 3P, and 4P were analyzed in both STRmix and EFM yielding a total of 1,257 of known-contributor LRs and 1,257 of known non-contributor LRs from each software.

The discrimination performance was evaluated qualitatively (Fig 2) and quantitatively (Fig 4) by checking the ability of each LR system in discriminating between H1-true and H2-true scenarios. The overall distribution plots (Fig 2) and ROC plots (Fig 4) suggest that the ability of the two LR systems to discriminate between known contributors and known non-contributors in aggregate are statistically indistinguishable for the data we considered.

Although both LR systems had similar discrimination performance, that did not imply that STRmix and EFM assigned equal LR values on a case-by-case basis even though LR computations were based on same/fixed EPG features, same pair of propositions, NOC, theta, and population allele frequency (Fig 5). The magnitude of differences was broken down into factor of 10 bins (Fig 6) and stratified by the type of POI (Fig 7). Differences in LR values greater than or equal to 3 on the log_10_ scale (as discussed in Section 3.6) were investigated and could occur due to one or more of the following reasons:

1. decisions made during parameters settings (e.g. choice of profiles for Model Maker interpretation and choice of settings for analysis such as analytical thresholds and drop-in parameters)
2. decision to analyze the same input files in both STRmix and EFM of which some of these profiles contained stutter peaks (F1 and B2) that were not modelled by EFM v2.1.0
3. non-convergence of the MCMC algorithms
4. differences in modelling assumptions of peak height information and variability, degradation, heterozygote balance, and allelic drop-outs/drop-ins

It is important to note that the apparent differences observed due to mentioned factors (2) and (3) were reduced upon re-interpretation of data both manually and in the software (e.g. re-interpreting profiles in EFM after removing the unmodelled F1 and B2 (S11 Table) or repeating analysis in STRmix with higher number of accepts (S8 Table).

Irrespective of the quantitative differences observed in certain cases between the LR systems (Fig 5), there seems to be a pattern observed in this study. Differences in LR values were observed in both directions (e.g., when LR STRmix ≥ 1000*LR EFM or when LR EFM ≥ 1000*LR STRmix). The magnitude of the differences was greater with minor donors than with equal or major contributors (Fig 7 and S11 and S12 Tables). Similar observations were documented in [34, 42, 62] when comparing LRs from various models.

Both LR systems showed adventitious exclusionary LR values (LR < 1) for H1-true tests (mainly with minor contributors) (Fig 3 and S6 Table) and adventitious inclusionary LR values (LR > 1) for H2-true tests (Fig 3 and S7 Table). The largest LR assigned using our LR systems and dataset was 587 from STRmix and 167 from EFM for a known unrelated non-contributor in the 1,257 H2-true tests (Fig 3C and S7 Table).

We observed that in certain cases differences in numerical LR values from both software resulted in differences in one or more than one verbal categories (Fig 8). These differences were substantially more with low template minor contributors and higher NOC (Fig 8 and S14 and S15 Tables); observations that have as well been examined in Swaminathan et al. [34]. Also, the cases of differences in the numerical LR values and verbal classification of the H2-true tests between the two models were higher than the ones observed with H1-true tests (Figs 6–8), thus showing the differences in the ability of both models to evaluate/measure the strength of evidence. The comparison of the assigned LR values in the verbal scale framework was included to provide some context to the observed differences. Although interesting, observed differences greater than 10^3^ may have less practical impact for large LRs (e.g. 10^15^ versus 10^18^) as compared to smaller LRs (e.g. 10^1^ versus 10^4^).

The findings of this study are specific to the LR systems (Fig 1) used in our study: (i) data chosen to generate parameter values and settings for analysis (e.g. Model Maker, analytical thresholds, drop-in, stutter settings), (ii) decisions made prior to the analysis of the mixture profiles in both software, and (iii) mixture profiles used for LR assessments. The profiles used for generating parameter values are shown in S1, S2, and S3 Tables. We also share with the forensic community the mixture profiles used for H1-true and H2-true tests with their corresponding LR values from both LR systems (S4 and S5 Tables). The comparisons performed in this study are more extensive than any software comparisons previously reported [34, 39–42, 54, 61, 62, 64, 68, 79]. The included supplementary tables and figures are intended to provide an example of the level of information and transparency we desire to see in similar DNA mixture publications. This provides the opportunity to review a specific mixture profile and further examine the assigned LR value(s). We believe that sharing the assigned LR values correlated with each mixture vs POI comparison complements the global aggregate level ROC and scatter plots used to assess the LR systems. This was further enabled by using the publicly available and consented PROVEDIt mixture profiles (i.e., the sharing of DNA profiles was not an issue). We encourage other investigators to assess the PROVEDIt profiles with *their* LR systems, compare their assigned LR values to those obtained in this study, and/or develop further visualization tools.

To sum up, “there are no true likelihood ratios, just like there are no true models” [80] and “no model perfectly incorporates all sources of uncertainty” [67]. The focus of this study is not to suggest that any one of the software is based on a true or best model. Our intent is to (i) understand the variability in LR values across different PG models, (ii) demonstrate the value of using a publicly available ground truth known mixture data [44] to assess performance of any LR system, (iii) describe how examining more than one PGS with similar discrimination power can be beneficial and an additional empirical diagnostic check even if software in use does contain certain diagnostic statistics as part of the output, (iv) share our observations with the forensic community that can lead to improving one or both models, and (v) address “Under what circumstances—and why—does the method produce results (random inclusion probabilities) that differ substantially from those produced by other methods?”, as recommended by the President’s Council of Advisors on Science and Technology (PCAST) report [81].

## Supporting information

S1 File Distribution of log_10_(LR) values for H1-true and H2-true tests of two, three, and four person mixtures by software and mixture ratios. (PPTX)

S2 File Distribution of log_10_(LR) values for H1-true and H2-true tests of two, three, and four person mixtures by software and varying DNA treatments. (PPTX)

S3 File Model examination. (PPTX)

S4 File An illustration of an example of a 2-person mixture profile of which LR (STRmix) > 1000*LR (EFM). (PPTX)

S5 File An illustration of an example of a 2-person mixture profile of which LR (EFM) > 1000*LR (STRmix). (PPTX)

S1 Table Single source sample profiles used in determining AT values. (XLSX)

S2 Table Negative control profiles used in determining drop-in parameters. (XLSX)

S3 Table Single source profiles included in the Model Maker analysis with varying range of DNA quality and quantity. (XLSX)

S4 Table Total H1-true calculations using mixtures with different ground truth number of contributors (NOC), total template amounts, type of POI (major, minor, and equal), and mixture ratios analyzed in both STRmix and EFM. (XLSX)

S5 Table Total H2-true calculations using mixtures with different ground truth number of contributors (NOC), total template amounts, and mixture ratios analyzed in both STRmix and EFM. (XLSX)

S6 Table Cases of 2P, 3P, and 4P mixture profiles with adventitious exclusionary LRs that resulted from H1-true tests in STRmix and EFM. (XLSX)

S7 Table Cases of 2P, 3P, and 4P mixture profiles with adventitious inclusionary LRs that resulted from H2-true tests in STRmix and EFM. (XLSX)

S8 Table Profile Log10(LRs) and per locus LRs of the H1-true tests that generated adventitious exclusionary LR values (LR = 0 or Log10(LR) = undefined) in STRmix when compared to known minor contributors due to a zero LR at a single locus. (XLSX)

S9 Table Profile Log10(LRs) of a 2P mixture profile that generated adventitious exclusionary LR value (LR < 1) in STRmix when compared to known minor contributor “42” due to artifact peaks retained in the input file. (XLSX)

S10 Table Diagnostics of Gelman-Rubin (GR) statistics. (XLSX)

S11 Table Overview of the H1-true calculations where LR (STRmix) ≥ 1000*LR (EFM). (XLSX)

S12 Table Overview of the H1-true calculations where LR (EFM) ≥ 1000*LR (STRmix). (XLSX)

S13 Table The SWGDAM verbal scale for the expression of the likelihood ratios. (XLSX)

S14 Table Verbal equivalents of the numeric LR values assigned by STRmix and EFM for the 2P, 3P, and 4P true contributor analysis (H1-true tests) based on the verbal convention recommendations set by the SWGDAM. (XLSX)

S15 Table Verbal equivalents of the numeric LR values assigned by STRmix and EFM for the 2P, 3P, and 4P true non-contributor analysis (H2-true tests) based on the verbal convention recommendations set by the SWGDAM. (XLSX)
