# Supplementary material for: Examining performance and likelihood ratios for two likelihood ratio systems using the PROVEDIt dataset
Source: PLoS One. 2021 Sep 17;16(9):e0256714. doi: 10.1371/journal.pone.0256714 (PMC8448353; doi:10.1371/journal.pone.0256714)
Supplement: S1 File — (PPTX) [file pone.0256714.s001.pptx]

## Slide 1
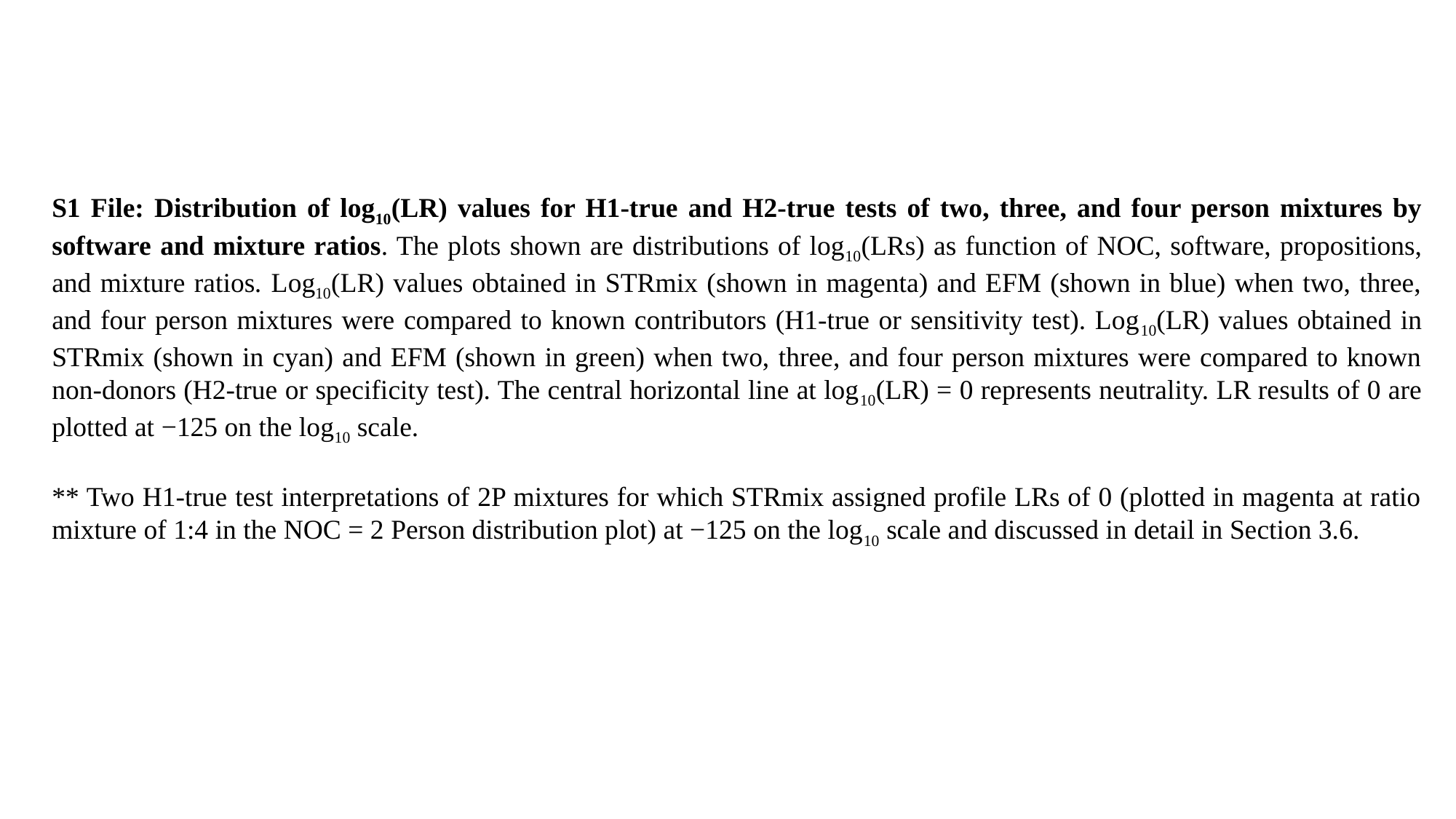

S1 File: Distribution of log10(LR) values for H1-true and H2-true tests of two, three, and four person mixtures by software and mixture ratios. The plots shown are distributions of log10(LRs) as function of NOC, software, propositions, and mixture ratios. Log10(LR) values obtained in STRmix (shown in magenta) and EFM (shown in blue) when two, three, and four person mixtures were compared to known contributors (H1-true or sensitivity test). Log10(LR) values obtained in STRmix (shown in cyan) and EFM (shown in green) when two, three, and four person mixtures were compared to known non-donors (H2-true or specificity test). The central horizontal line at log10(LR) = 0 represents neutrality. LR results of 0 are plotted at −125 on the log10 scale.
** Two H1-true test interpretations of 2P mixtures for which STRmix assigned profile LRs of 0 (plotted in magenta at ratio mixture of 1:4 in the NOC = 2 Person distribution plot) at −125 on the log10 scale and discussed in detail in Section 3.6.

## Slide 2
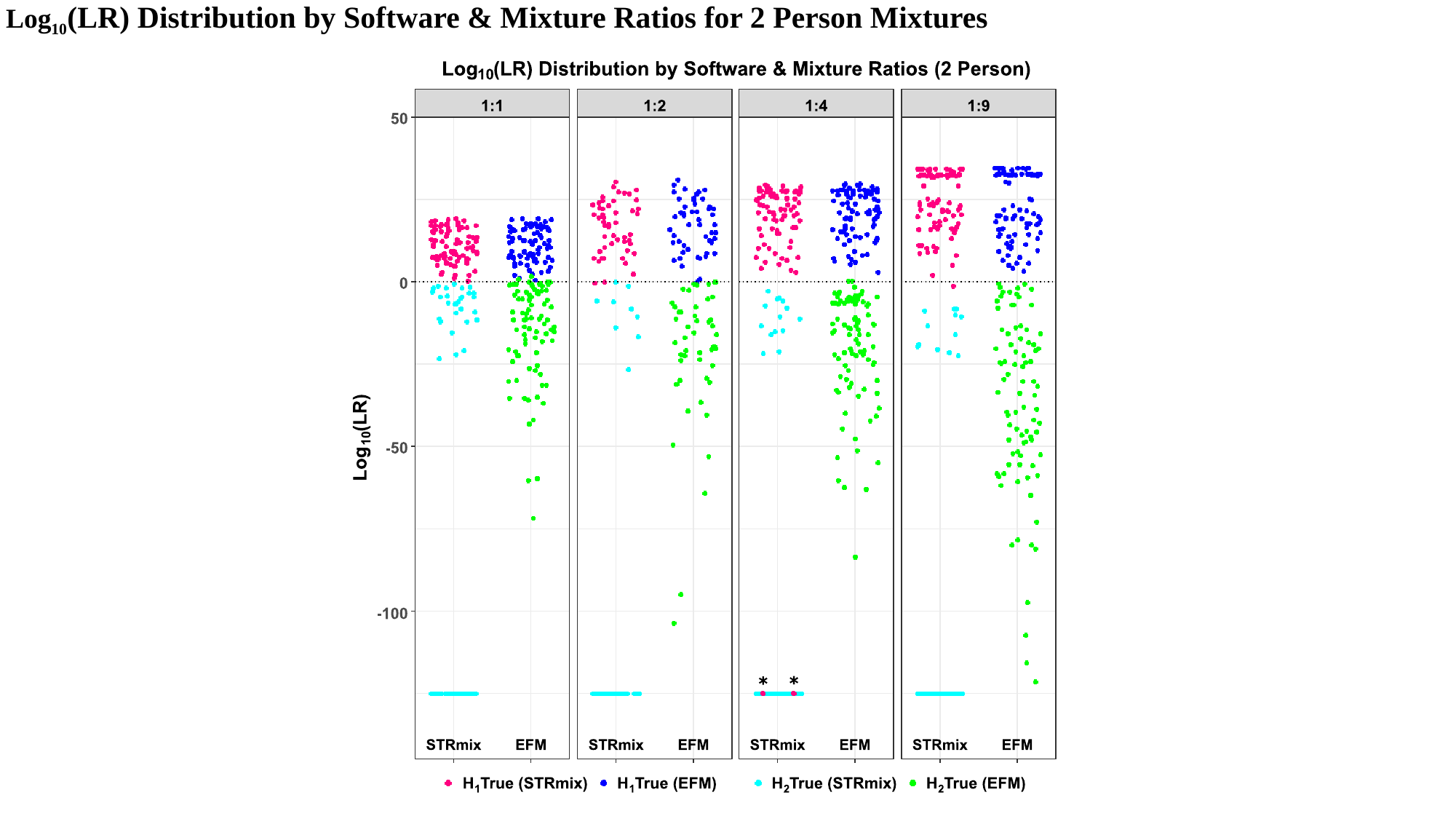

Log10(LR) Distribution by Software & Mixture Ratios for 2 Person Mixtures

## Slide 3
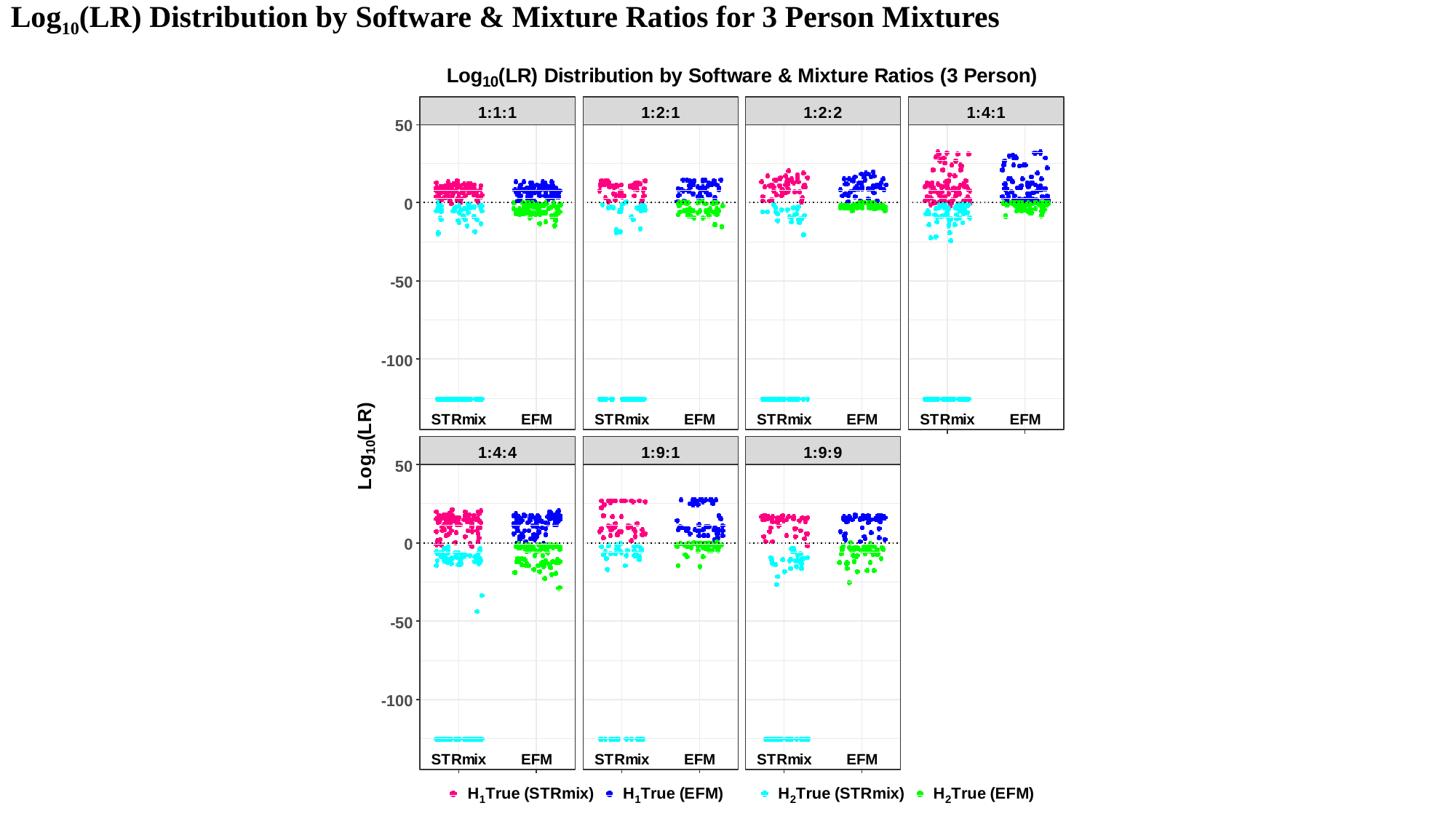

Log10(LR) Distribution by Software & Mixture Ratios for 3 Person Mixtures

## Slide 4
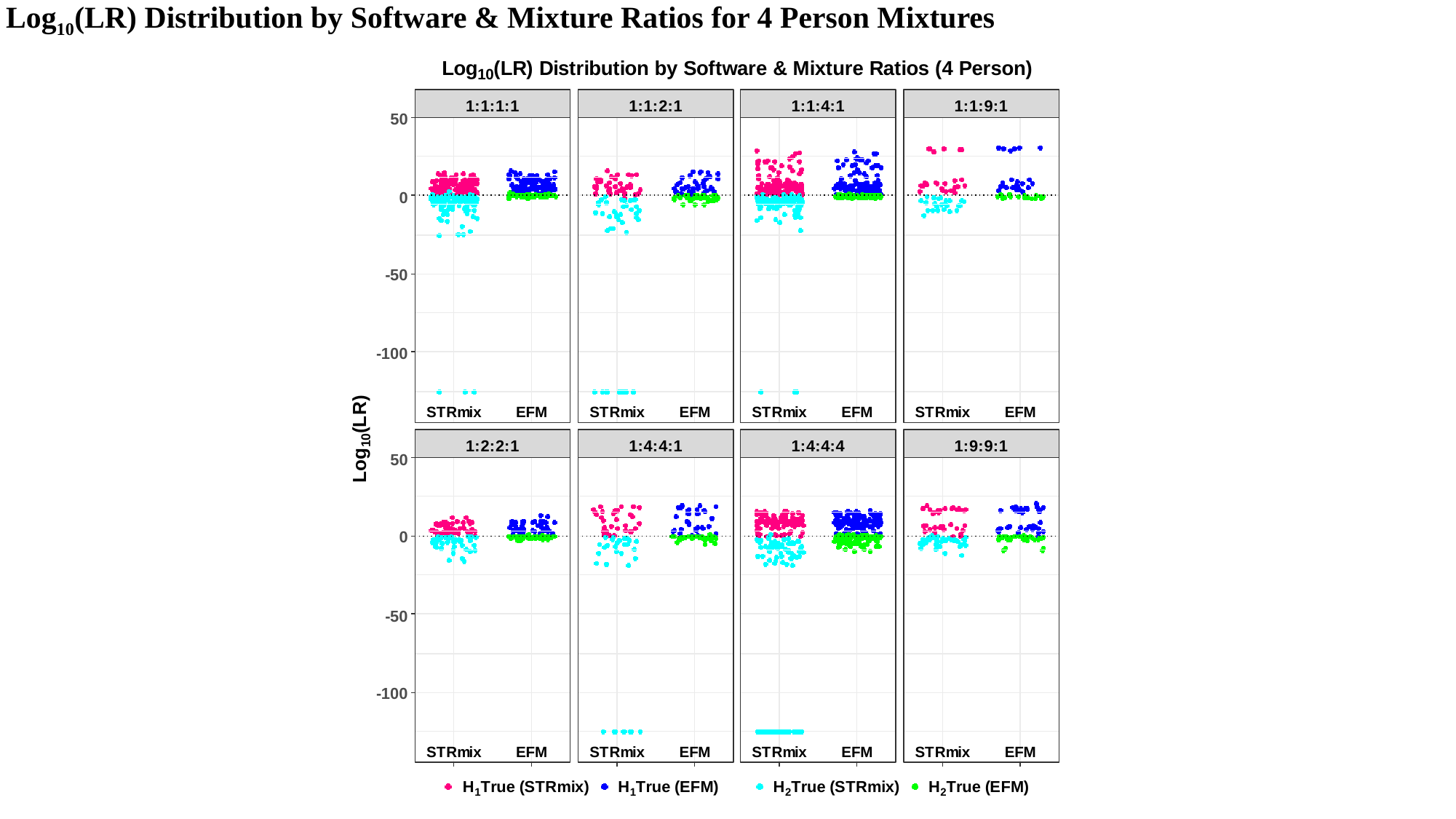

Log10(LR) Distribution by Software & Mixture Ratios for 4 Person Mixtures
