# Supplementary material for: Examining performance and likelihood ratios for two likelihood ratio systems using the PROVEDIt dataset
Source: PLoS One. 2021 Sep 17;16(9):e0256714. doi: 10.1371/journal.pone.0256714 (PMC8448353; doi:10.1371/journal.pone.0256714)
Supplement: S2 File — (PPTX) [file pone.0256714.s002.pptx]

## Slide 1
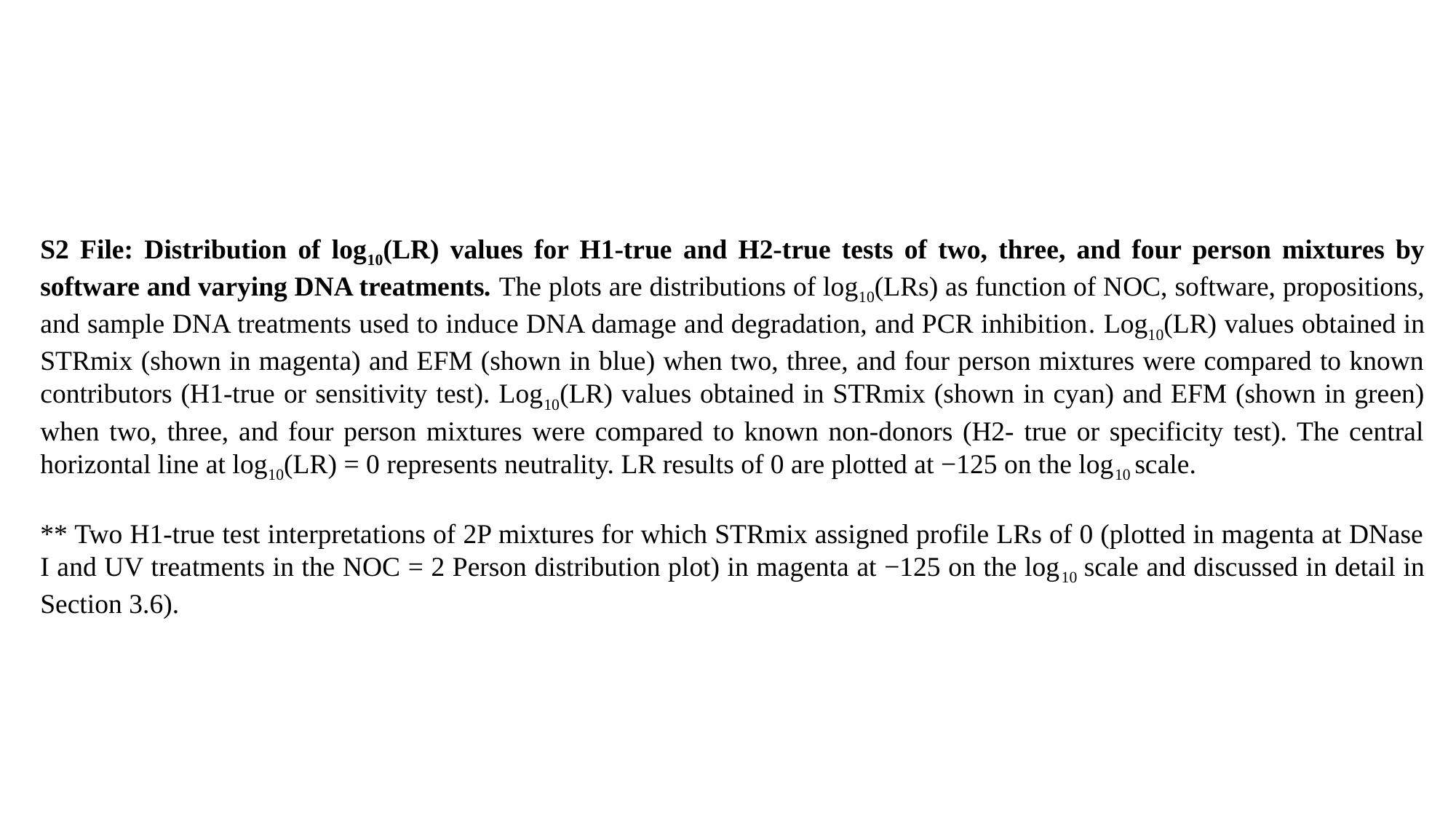

S2 File: Distribution of log10(LR) values for H1-true and H2-true tests of two, three, and four person mixtures by software and varying DNA treatments. The plots are distributions of log10(LRs) as function of NOC, software, propositions, and sample DNA treatments used to induce DNA damage and degradation, and PCR inhibition. Log10(LR) values obtained in STRmix (shown in magenta) and EFM (shown in blue) when two, three, and four person mixtures were compared to known contributors (H1-true or sensitivity test). Log10(LR) values obtained in STRmix (shown in cyan) and EFM (shown in green) when two, three, and four person mixtures were compared to known non-donors (H2- true or specificity test). The central horizontal line at log10(LR) = 0 represents neutrality. LR results of 0 are plotted at −125 on the log10 scale.
** Two H1-true test interpretations of 2P mixtures for which STRmix assigned profile LRs of 0 (plotted in magenta at DNase I and UV treatments in the NOC = 2 Person distribution plot) in magenta at −125 on the log10 scale and discussed in detail in Section 3.6).

## Slide 2
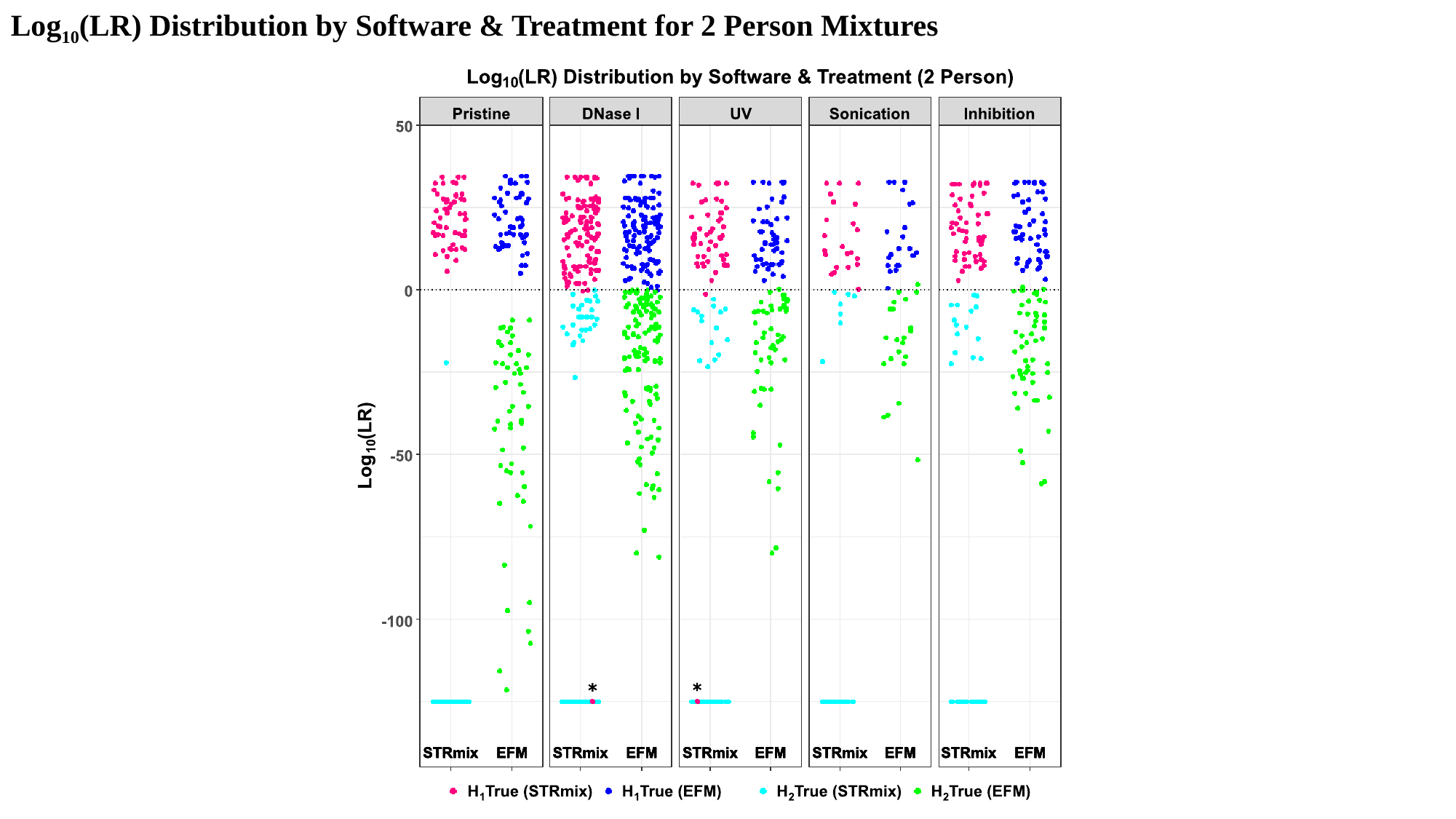

Log10(LR) Distribution by Software & Treatment for 2 Person Mixtures

## Slide 3
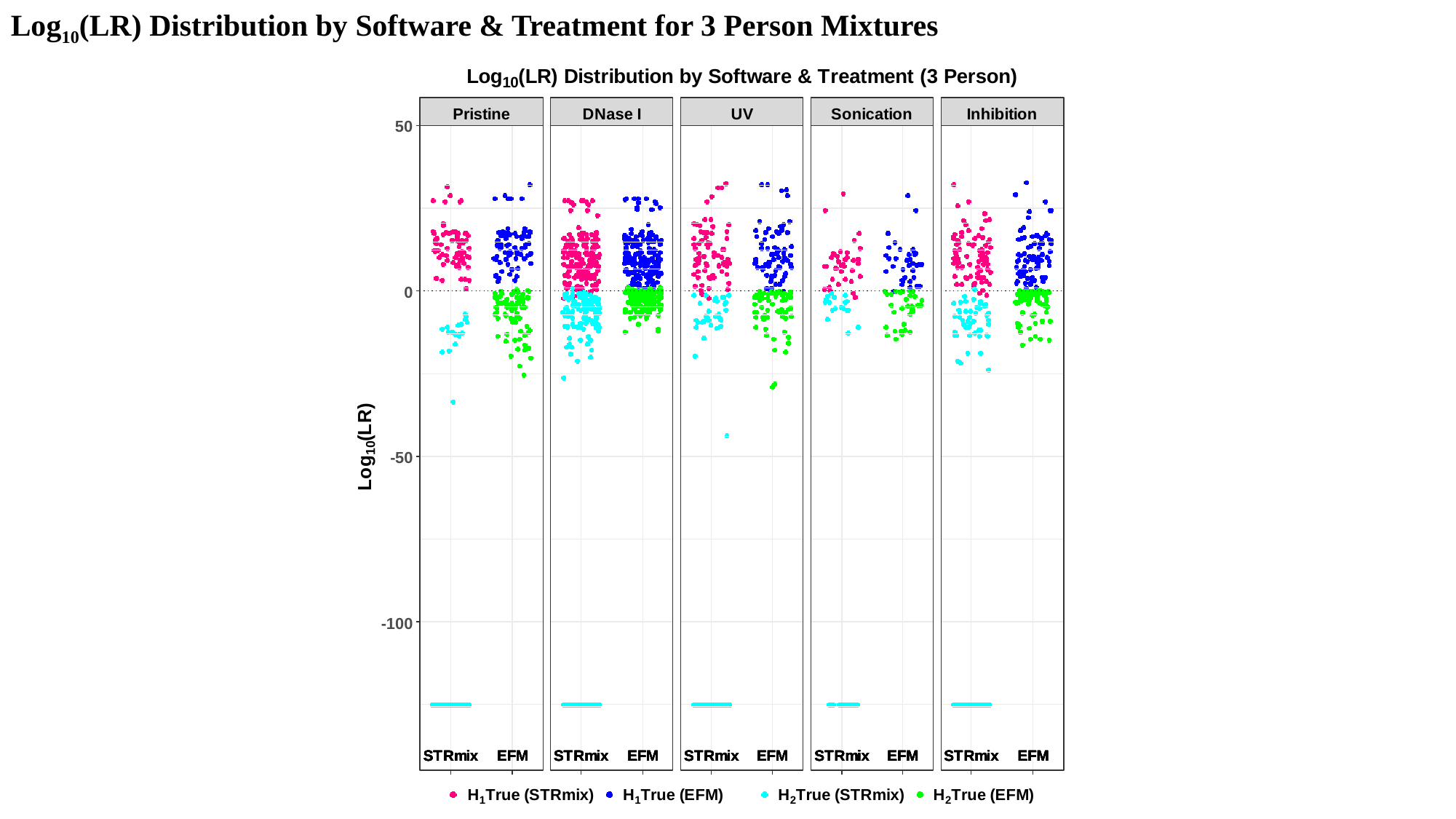

Log10(LR) Distribution by Software & Treatment for 3 Person Mixtures

## Slide 4
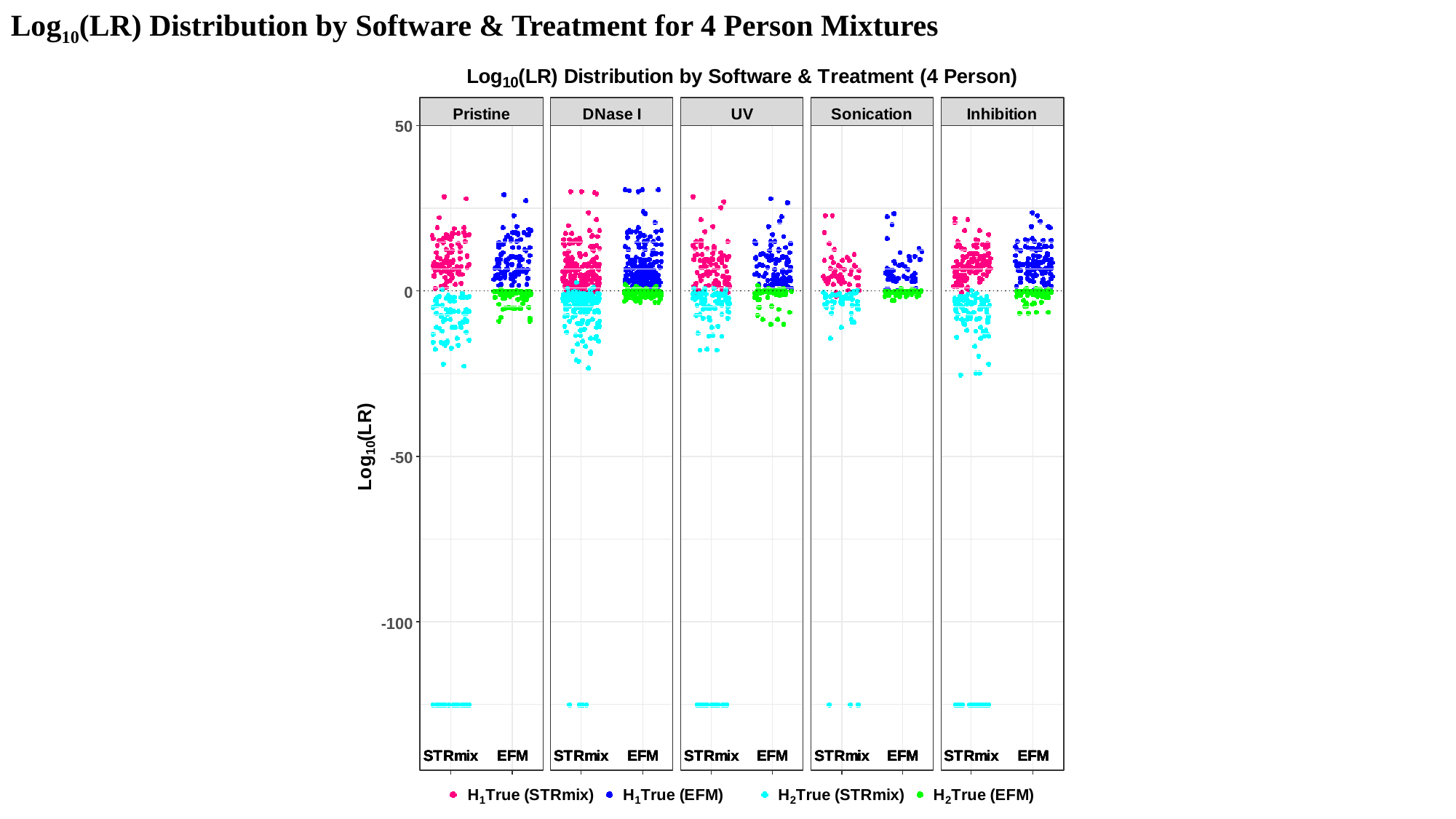

Log10(LR) Distribution by Software & Treatment for 4 Person Mixtures
